# The DNA Demethylase TET1 is a Pivotal Regulator of the miR-124/ISX9-Instructed Conversion of Astrocytes to Induced Neurons

**DOI:** 10.1007/s12035-026-05873-1

**Published:** 2026-04-25

**Authors:** Elsa Papadimitriou, Lukas daCC Iohan, Alexandra Frazeskou, Evangelia Xingi, Marcos R. Costa, Dimitra Thomaidou

**Affiliations:** 1 Neural Stem Cells and Neuroimaging Group, Department of Neurobiology, Hellenic Pasteur Institute, Athens, Greece; 2 Brain Institute, Federal University of Rio Grande Do Norte, Natal, Brazil; 3 Bioimaging Unit, Hellenic Pasteur Institute, Athens, Greece; 4 Roche Pharma Research and Early Development, Neuroscience and Rare Diseases, Roche Innovation Center Basel, F. Hoffmann-La Roche Ltd, Basel, Switzerland

**Keywords:** Astrocytes, Direct reprogramming, iR-124, ISX9, Transcriptional regulatory network, TET1, LIN28A

## Abstract

The microRNA miR-124 promotes neuronal identity by acting globally, through its multiple targets, at the epigenetic, transcriptional and post-transcriptional levels. We have previously shown that miR-124 acts as a potent driver of the astrocytic fate switch toward an immature neuronal identity, while its supplementation with the neurogenic compound ISX9 enhances in vitro neuronal maturation. Nevertheless, additional cues are needed to enhance the in vivo neurogenic reprogramming capacity of miR-124/ISX9 following neurodegeneration or neurotrauma. In this study, we constructed the core transcriptional regulatory network regulated by miR-124 and ISX9 during astrocyte-to-neuron conversion. Our analysis revealed that the DNA demethylase TET1 is a pivotal transcriptional regulator of the miR-124/ISX9 neurogenic reprogramming process. Silencing of *Tet1* impaired the miR-124-mediated neuronal conversion of astrocytes, as well as the ISX9-reinforced differentiation of iNs. We also identified the DNA/RNA binding protein LIN28A as the top mediator of ISX9 neurogenic action and provide evidence that it acts as a coregulator of the expression of synaptic genes, along with TET1. Taken together, our data suggest that TET1 and LIN28A are potent candidates for amplifying miR-124/ISX9 combined in vivo reprogramming action and enhance iNs’ differentiation state.

**Supplementary Information:**

The online version contains supplementary material available at 10.1007/s12035-026-05873-1.

## Introduction

Direct neurogenic reprogramming of astrocytes is a powerful regenerative approach with therapeutic promise, given the high abundance and neural stem cell (NSC) potential of brain resident astrocytes [1, 2]. To this end, the conversion of astrocytes to induced neurons (iNs) has been well-studied in vitro and in vivo using combinations of neurogenic transcription factors (TFs), miRNAs and chemical cocktails [3–12]. Nevertheless, a number of studies agree that the efficiency of astrocyte-to-neuron conversion in vivo remains lower than in vitro and iNs remain primarily immature [13, 14], thus overcoming barriers to improve both neurogenic transition and iNs’ maturation is necessary before progressing to a therapeutic direction.

miRNAs have been successfully employed in various reprogramming protocols for the production of iNs [6, 14–16], induced cardiomyocytes [17, 18] and induced pluripotent stem cells [19, 20], due to their capacity to fine-tune a wide range of lineage specific genes and epigenetic regulators and thus repress the initial cell fate [21–24]. Among neurogenic miRNAs, the combination of miR-124 and miR-9/9* has been used for the generation of iNs, primarily from fibroblasts and, more recently, from astrocytes, pericytes and smooth muscle cells, either alone [24, 25] or in combination with neurogenic TFs [6, 15, 26], whereas miR-124 has been combined with the neurogenic compound ISX9 [14]. Additionally, the less well-studied miRNAs miR-218 and miR-34b/c have been used only in combination with lineage-specific TFs [16, 27].

The brain-enriched miRNA, miR-124, acts globally to promote neuronal fate by impacting gene expression through its pivotal targets at the epigenetic, transcriptional and post-transcriptional levels. More specifically, miR-124 has been shown to increase the expression of neuronal genes by repressing components of major neuronal gene repressor complexes, such as the phosphatase of the REST complex *Scp1* [28] and the histone methyltransferase of the PRC2 complex *Ezh2* [29]. It also impacts the post-transcriptional regulation of neuronal transcripts by targeting the global repressor of neuron-specific splicing *Ptbp1* [30] and the RNA binding protein *Zfp36l1*, which is implicated in the mRNA decay of neuron-specific transcripts [14]. In addition to its roles in transcriptional and post-transcriptional regulation, miR-124 also contributes to a chromatin permissive environment for neuronal reprogramming through its involvement in the formation of the neuron-specific chromatin remodeling complex nBAF by targeting the non-neuronal component *Baf53a* and promoting its exchange with the neuronal specific BAF53B [22].

We have previously shown that miR-124 is an efficient driver of the cell fate switch of mouse cortical astrocytes toward an immature neuronal identity in vitro and that the addition of the neurogenic compound ISX9 leads to iNs’ differentiation and functional maturation. We have also shown that miR-124 is potent in guiding the direct conversion of reactive astrocytes to immature iNs in vivofollowing cortical trauma, whereas ISX9 administration confers a survival benefit to newly generated iNs [14]. However, the synergistic action of miR-124/ISX9 in vivo seems to be less efficient thanin vitro in driving astrocyte-to-neuron conversion and iNs’ differentiation, indicating that more intrinsic and/or extrinsic cues are needed. To this end, the identification of the core downstream effectors of miR-124 and ISX9 that have the capacity to amplify the combined effects of miR-124/ISX9 in vitro reprogramming action and enhance iNs’ production and differentiation is a prerequisite for the subsequent testing of their in vivo efficacy to amplify miR124/ISX9 action and sufficiently induce neurogenic reprogramming of astrocytes following brain injury and neurodegeneration.

In this study, we sought to identify the transcriptional regulators enacted by miR-124 and ISX9 to instruct the neurogenic conversion of astrocytes, aiming to reveal novel core neurogenic factors with instrumental role in the reprogramming process. By constructing a transcriptional regulatory network (TRN) regulated by miR-124 alone or in collaboration with ISX9 and inferring the core transcriptional regulators in this network, we identified the DNA demethylase TET1 as a pivotal reprogramming regulator under both conditions and revealed that the silencing of *Tet1* strongly impacts the early neuronal conversion of both miR-124-iNs and miR-124 + ISX9-iNs and significantly hampers the differentiation of miR-124 + ISX9-iNs. We further showed that the DNA/RNA binding protein LIN28A, identified in our TRN analysis among the top ISX9-controlled regulators, plays an important role in the morphological differentiation of miR-124 + ISX9-iNs. Importantly, we provide evidence that LIN28A and TET1 coregulate a set of genes that contribute to neuronal differentiation, among which genes with synaptic roles.

## Results

### TRN Analysis Identifies Core Transcriptional Regulators in miR-124- and miR-124/ISX9-Instructed Reprogramming

**Fig. 1:**
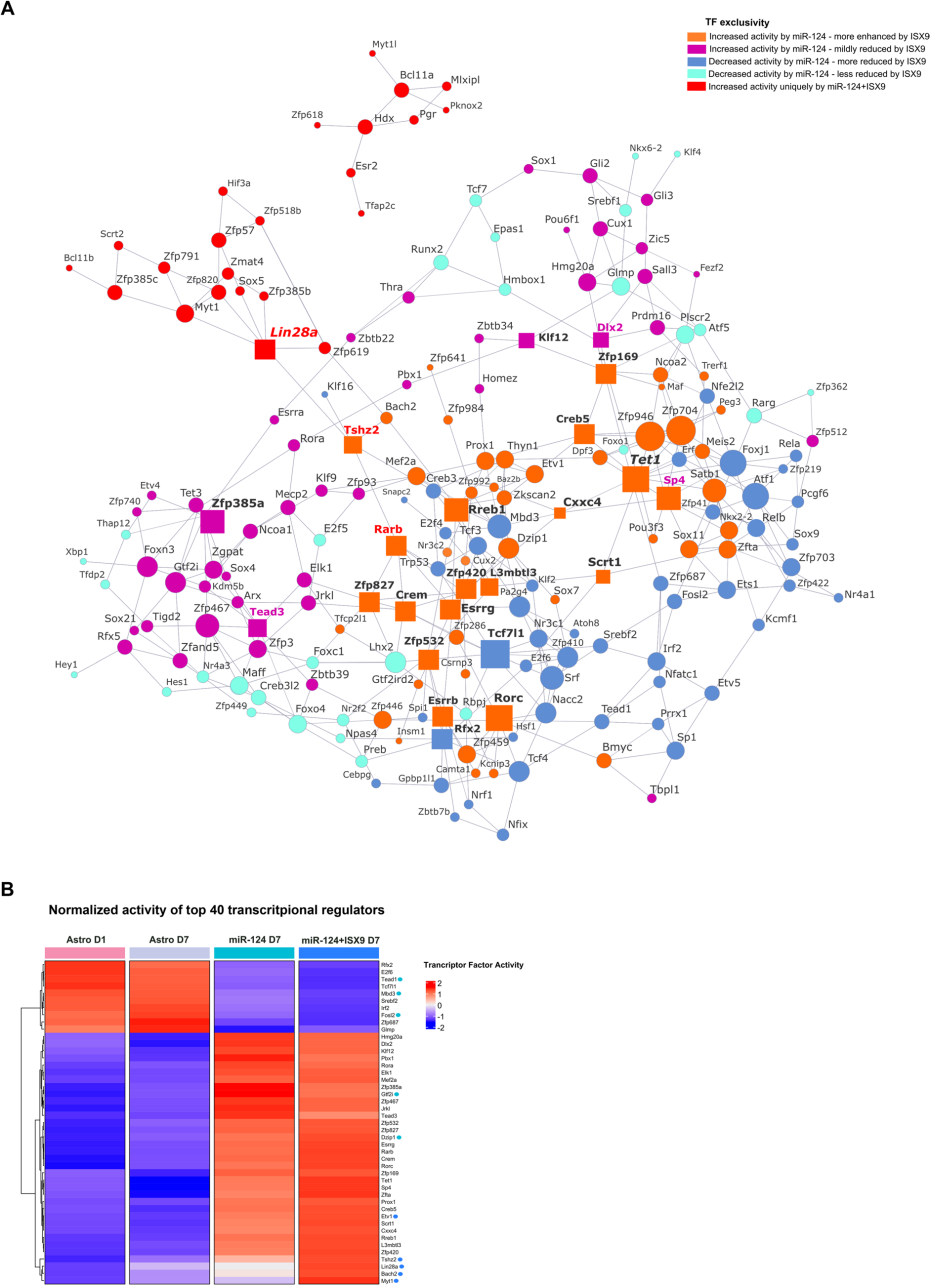
TRN analysis identifies core transcriptional regulators with pivotal roles in miR-124 and miR-124/ISX9 instructed reprogramming. **A.** Representation of the TRN constructed from RNA-seq data obtained from astrocytes at days 1 and 7 of reprogramming, miR-124-reprogrammed astrocytes on day 7 and miR-124 + ISX9-reprogrammed astrocytes on day 7. Each transcriptional regulator is represented by a circle (or a box for the top 20 TFs), where its size is proportional to its regulon size, and its color is indicative of its activity induced by miR-124 and modified by ISX9 (**Orange:** transcriptional regulators whose activity is increased by miR-124 and further enhanced by ISX9 addition (miR-124 + ISX9 > miR-124 > astro day 1/day 7)). **Magenta**: transcriptional regulators whose activity is increased by miR-124 and mildly reduced by ISX9 (miR-124 > miR-124 + ISX9 > astro day 1/day 7). **Red:** transcriptional regulators whose activity is uniquely increased by the action of ISX9. **Blue:** transcriptional regulators whose activity is reduced by the action of miR-124 and further reduced by ISX9 (astro day 1/day 7 > miR-124 > miR-124 + ISX9). **Turquoise:** transcriptional regulators whose activity is reduced by miR-124 and less reduced by ISX9 (astro day 1/day 7 > miR-124 + ISX9 > miR-124). The top 20 transcriptional regulators inferred from the betweenness centrality analysis of the TRN of miR-124 and miR-124 + ISX9 are also portrayed in this network as boxes, and their names are highlighted in bold and black for common regulators, magenta for unique regulators in miR-124 condition and red in miR-124 + ISX9 condition. **B.** Heatmap presenting the normalized activity of the top 40 transcriptional regulators for miR-124 and miR-124 + ISX9 conditions inferred via betweenness centrality analysis of the TRN (35 regulons are common, whereas the unique 5 regulons for each condition are indicated by green and blue circles for miR-124 and miR-124 + ISX9, respectively)

To identify the master transcriptional regulators that are modulated during the reprogramming of primary mouse astrocytes into immature iNs by miR-124 and functional iNs by miR-124 along with ISX9, we leveraged our previously published RNA-seq data obtained from astrocytes and lineage-converted iNs after 7 days in vitro [14] to generate a transcriptional regulatory network (TRN) inferred from TF‒gene interactions (hereafter referred to as regulons) using the RTN and ARACNe R packages [31, 32]. We identified 279 regulons with different degrees of activity in astrocytes (obtained on day 1 and day 7), miR-124-iNs and miR-124 + ISX9-iNs (Fig. 1A and Suppl. File 1). As expected, we observed a strong impact of miR-124 treatment on the astrocytic transcriptome, conferring a reduction in the activity of TFs enriched in astrocytes, such as LHX2 [33], NR3C1 [34], SP1 [35], RUNX2 [36], SOX9 [37], TEAD1 [38], RFX2 and TCF7L1 [39] (in blue and turquoise), and a parallel increase in the activity of neurogenic transcriptional regulators, such as CREM [40], CUX1 [41], DLX2 [42], MEF2A [43], MECP2 [44], RORC [45], RREB1 [46], SCRT1 [47], SOX11 [48], SP4 [49] and TET1 [50] (in orange and violet). Cotreatment with ISX9 reinforced part of this regulatory network by further increasing the activity of neurogenic transcriptional regulators activated by miR-124 (in orange) and further reducing the activity of astrocytic regulators repressed by miR-124 (in blue). Importantly, the astrocyte-to-neuron conversion mediated by the combination of miR-124 with ISX9 induced a unique set of genes regulated by well-characterized neurogenic transcriptional regulators, such as BCL11A [51], LIN28A [52], MYT1 [53], MYT1L [54] and SCRT2 [47] (in red).

Next, we sought to identify the key regulators in this network by calculating the betweenness centrality of the TRN, which is a measure of network centrality that estimates the bridgeness of a node and its importance in controlling the flow and stability of the network [55, 56] (Suppl. File 2). The normalized activity of the top 40 transcriptional regulators in our TRN, identified by betweenness centrality analysis, is presented in Fig. 1B, while the top 20 regulators (Table 1) are highlighted in the TRN in boxes (17 common and 3 unique regulators for each condition: SP4, DLX2 and TEAD3 for miR-124 condition and LIN28A, TSHZ2 and RARB for miR-124 + ISX9 condition). Interestingly, TCF7L1 and RFX2 were the only downregulated TFs in the top 20 list of both conditions (shown in blue boxes in Fig. 1A). Importantly, when we compared the regulons of miR-124 and miR-124 + ISX9 conditions with the regulons of ASCL1- and NEUROG2-induced astrocyte-to-neuron conversion [57], we observed that the regulatory network established by miR-124/ISX9 shares several common regulons with the network of ASCL1 (Suppl. Figure 1A) and, to a lesser extent, with that of NEUROG2 (Suppl. Figure 1B).

**Table 1:** Top 20 regulons (transcriptional regulator: gene target interactions) related to the miR-124 and miR-124 + ISX9 conditions inferred via betweenness centrality analysis of the TRN

miR-124		miR-124 + ISX9	
Gene	Betweenness centrality score	Gene	Betweenness centrality score
Zfp169	1651.034811	Tshz2	2327.333197
Scrt1	1637.724484	Lin28a	2112.539834
Tet1	1574.66004	Rarb	1942.673477
Cxxc4	1300.680237	Zfp169	1920.969504
Rfx2	1176.812181	Scrt1	1824.766249
L3mbtl3	1175.419131	Tet1	1728.65451
Zfp532	1155.891175	Rreb1	1702.456153
Rorc	1155.703736	Cxxc4	1493.545635
Zfp420	1145.904726	L3mbtl3	1469.627762
Tcf7l1	1121.553988	Crem	1367.485143
Zfp385a	1110.984518	Tcf7l1	1359.29953
Klf12	1065.056285	Zfp420	1351.200111
Crem	976.762115	Zfp532	1288.4918
Sp4	920.8416381	Rorc	1283.265023
Creb5	917.3332295	Zfp827	1270.328126
Tead3	903.740104	Rfx2	1241.35441
Rreb1	898.5286039	Creb5	1232.523292
Zfp827	867.6912943	Zfp385a	1211.125189
Dlx2	859.3121672	Klf12	1119.332765
Esrrg	853.7662553	Esrrg	1119.229133

Our analysis revealed that TET1 was among the top 10 regulators, active both in miR-124-iNs (top 3) and in miR-124 + ISX9-iNs (top 6) (Table 1), suggesting a key role for this transcriptional regulator in the reprogramming process instructed by miR-124/ISX9. Interestingly, TET1 was found to be shared among miR-124/ISX9-, ASCL1- and NEUROG2-regulated TRNs, supporting its global role in astrocyte-to-neuron conversion (Suppl. Figure  1 A and B). Therefore, we chose TET1 for further study, as it is a well-known regulator of DNA methylation during neurogenesis [50, 58, 59], whereas its role in neurogenic reprogramming has been previously reported, but not studied in depth [26, 60].

### *Tet1* silencing greatly reduces the reprogramming efficiency and early differentiation of both miR-124-iNs and miR-124 + ISX9-iNs

**Fig. 2:**
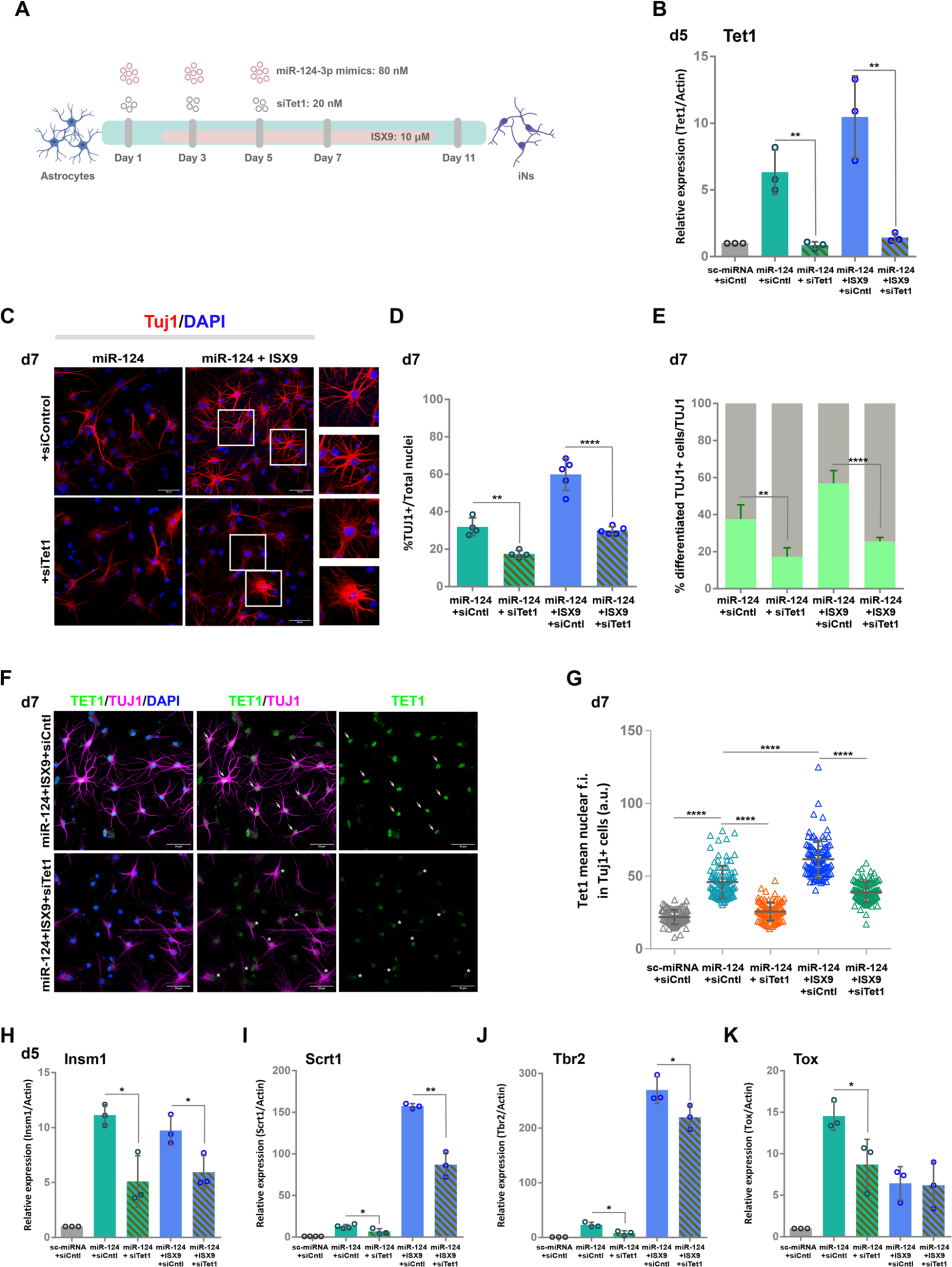
*Tet1* knockdown greatly reduces the reprogramming efficiency and early differentiation of both miR-124-iNs and miR-124 + ISX9-iNs. **A.** Schematic representation of the protocol used to silence *Tet1* during the reprogramming process. **B.** Estimation of the degree of *Tet1* mRNA silencing on day 5 of reprogramming by miR-124 -/+ siTet1 or miR-124 + ISX9 -/+ siTet1 via qRT‒PCR. n = 3 independent experiments. **C.** Immunostaining of astrocytes reprogrammed with miR-124 -/+ siTet1 or miR-124 + ISX9 -/+ siTet1 on day 7 with an anti-TUJ1 antibody (in red); the inset areas show representative cell morphologies. **D.** Quantification of the percentage of TUJ1 + reprogrammed cells with miR-124 -/+ siTet1 (n = 4 independent experiments) or miR-124 + ISX9 -/+ siTet1 (n = 5 independent experiments) on day 7. **E.** Presentation of the proportion of differentiated TUJ1 + iNs (green portions of the bars) in the total TUJ1 + population (as quantified in **D** for each condition and set to 100%) (the gray portions of the bars indicate the proportion of TUJ1 + iNs exhibiting a transitory still not differentiated morphology). **F.** Immunostaining of astrocytes reprogrammed with miR-124 + ISX9 -/+ siTet1 on day 7 with an anti-TUJ1 antibody (in magenta) and an anti-TET1 antibody (in green); representative cells exhibiting high TET1 levels and a differentiated morphology (categories 1 or 2) are indicated with arrows, while representative cells with low TET1 levels and a less differentiated morphology (categories 2 or 3) are indicated with asterisks. **G.** Measurement of the mean nuclear fluorescence intensity (f.i.) of TET1 protein levels on day 7 in control cells treated with sc-miRNA + si-Cntl and TUJ1 + reprogrammed cells treated with miR-124 -/+ siTet1 or miR-124 + ISX9 -/+ siTet1 (n = 100 cells for each condition from 3 independent experiments) (mean ± SEM, p < 0.0001, two tailed t-test assuming unequal variance).RT‒qPCR analysis of the mRNA levels of the TFs related to early neurogenesis *Insm1* (**H**), *Scrt1* (**I**), *Tbr2* (**J**) and *Tox* (**K**) in astrocytes reprogrammed with miR-124 -/+ siTet1 or miR-124 + ISX9 -/+ siTet1 on day 5. n = 3 independent experiments. *p < 0.05, **p < 0.01, ****p < 0.0001

To experimentally evaluate the role of TET1 in the reprogramming process instructed by miR-124 and miR-124 + ISX9, we silenced *Tet1* in control astrocytes and in miR-124- and miR-124 + ISX9-overexpressing astrocytes using a pool of 4 siRNAs specific for mouse *Tet1* mRNA, following the protocol presented in Fig. 2A. Treatment of astrocytes with siTet1 led to a significant reduction in *Tet1* mRNA levels on day 5 of reprogramming both in miR-124 and miR-124 + ISX9 conditions (86.2% ± 3.5% and 86.1% ± 2.0%, respectively), as estimated by qRT‒PCR (Fig. 2B). Similar silencing efficiency was achieved on day 7 in both conditions (Suppl. Figure  2 A). We also verified that *Tet1* silencing did not affect the mRNA levels of the paralogous genes *Tet2* and *Tet3* (Suppl. Figure 2B and C).

First, we sought to estimate the impact of *Tet1* silencing on early reprogramming induced by both miR-124 and miR-124 + ISX9 by immunofluorescently labeling cells for the neuronal-specific cytoskeletal marker beta-IΙΙ-TUBULIN (TUJ1, red) (Fig. 2C and Suppl. Figure 2D). To estimate the number of TUJ1 + cells undergoing reprogramming, a set of morphological rules was applied in addition to their positivity for TUJ1. More specifically, TUJ1 + cells were divided into four groups according to their soma size and their morphology (for a detailed analysis see Materials and Methods). Briefly, TUJ1 + cells with a small soma area ranging from 85–200 a.u. (category 1, representative cells are presented in Suppl. Figure  3 A) or 200–500 a.u. (category 2, representative cells are presented in Suppl. Figure 3B), where all (category 1) or most (category 2) of their primary neurites are well-defined were considered as progressing efficiently in the reprogramming process exhibiting a differentiated neuronal-like morphology. On the other hand, cells with a bigger soma area ranging from 500–1000 a.u (category 3, representative cells are presented in Suppl. Figure  3 C) harboring many not well-defined primary neurites were considered as still undergoing reprogramming, however less successfully, harboring a transitory morphology. Lastly, TUJ1 + cells with an astrocyte-like morphology characterized by a very large soma area ranging from 1000–1600 a.u. (category 4, representative cells are presented in Suppl. Figure 3D) with few or no primary neurites were excluded from the analysis, considered to have failed to reprogram. On the basis of this morphological classification, we estimated that silencing *Tet1* significantly reduced the percentage of all TUJ1 + cells undergoing reprogramming by 44.2% (from 28.0% ± 7.8% to 15.6% ± 0.7%) in miR-124 condition and 55.3% (from 42.6% ± 5.6% to 19.0% ± 9.5%) in miR-124 + ISX9 condition on day 5 (Suppl. Figure 2D and E) and by 49.0% (from 32.0% ± 4.7% to 16.3% ± 1.1) in miR-124 condition and 50.7% (from 60.0% ± 8.5% to 29.6% ± 2.8%) in miR-124 + ISX9 condition on day 7 (Fig. 2C and 2D). Additionally, apart from reducing the number of TUJ1 + cells undergoing reprogramming, *Tet1* knockdown impeded morphological changes that accompany iN differentiation, resulting in an even greater reduction in the number of TUJ1 + cells with differentiated morphology both on day 5 (Suppl. Figure 2D and F) and on day 7 (Fig. 2C and 2E, insets in Fig. 2C show representative morphologies). Quantification of reprogrammed cells on the basis of their morphology revealed that the number of TUJ1 + cells harboring a differentiated morphology was reduced by 70.1% (from 22.2% ± 9.8% to 6.6% ± 2.7%) and 71.1% (from 39.3% ± 12.6% to 11.4% ± 6.4%) in miR-124 and miR-124 + ISX9 conditions, respectively, on day 5 (Suppl. Figure  2 F), and by 44.5% (from 37.6% ± 7.7.7% to 17.3% ± 4.8%) and 57.6% (from 56.9% ± 6.9% to 25.6.0% ± 2.1%) in miR-124 and miR-124 + ISX9 conditions, respectively, on day 7 (Fig. 2E). Importantly, measuring the mean fluorescence intensity (f.i.) of TET1 in the nuclei of miR-124 (Suppl. Figure 2G and Fig. G) and miR-124 + ISX9 (Fig. 2F and G) reprogrammed TUJ1 + cells on day 7, we verified the reduction of TET1 at the protein level and correlated the low TET1 protein levels with the low differentiation status of TUJ1 + cells that undergo reprogramming. Furthermore, qRT‒PCR analysis on day 5 in a set of neurogenic TFs with role early in neurogenesis, namely, *Ascl1*, *Tox*, *Insm1*, *Tbr2*, and *Scrt1* (shown in our previous work to be upregulated by miR-124 and further enhanced for the latter two by ISX9), further indicated that silencing *Tet1* resulted in a significant downregulation of the mRNA levels of *Insm1* by 55.0% ± 17.0% and 39.4% ± 8.0% in miR-124 and miR-124 + ISX9 conditions, respectively (Fig. 2H), *Scrt1* by 48.6% ± 14.8% and 44.8% ± 14.2% (Fig. 2I) and *Tbr2* by 62.0% ± 23.2% and 19.5% ± 8.3% (Fig. 2J), while it significantly decreased *Tox* by 40.5% ± 18.4% only in miR-124 condition (Fig. 2K), whereas it did not significantly affect the mRNA levels of *Ascl1* (Suppl. Figure 2H). Importantly, *Scrt1* has been identified as part of the TET1 regulon, which comprises 118 genes (Suppl. File 3).

The above findings indicate that TET1 plays a crucial role in miR-124 early instructed reprogramming, as well as in the neurogenic conversion and early differentiation of miR-124 + ISX9-iNs, verifying its presence in the top 10 transcriptional regulator lists of both networks.

### TET1 is Important for the Differentiation of miR-124 + ISX9-iNs and Controls Key Regulators of Neurogenic Reprogramming

**Fig. 3:**
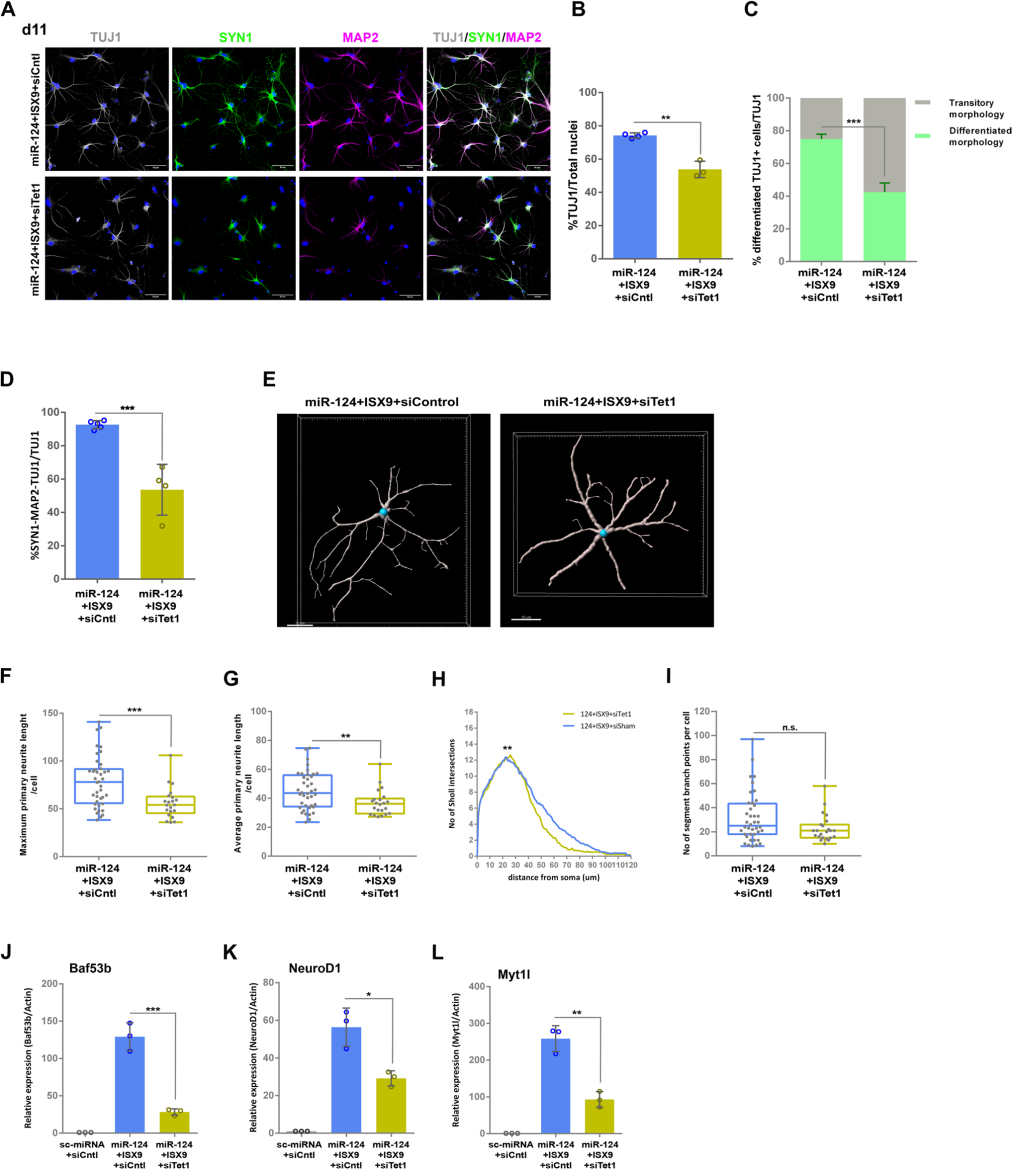
TET1 is important for the neuronal differentiation of miR-124 + ISX9-iNs and controls key regulators of neurogenic reprogramming. **A.** Coimmunostaining of astrocytes reprogrammed with miR-124 + ISX9 -/+ siTet1 on day 11 with anti-TUJ1 (in gray), anti-SYN1 (in green) and anti-MAP2 (in magenta) antibodies. **B.** Quantification of the percentage of TUJ1 + reprogrammed cells with miR-124 + ISX9 + siCntl (n = 4 independent experiments) or miR-124 + ISX9 + siTet1 (n = 3 independent experiments) on day 11. **C.** Presentation of the proportion of differentiated TUJ1 + iNs (green portions of the bars) in the total TUJ1 + population (as quantified in **B** for each condition and set to 100%) (the gray portions of the bars indicate the proportion of TUJ1 + iNs exhibiting a transitory still not differentiated morphology). **D.** Quantification of the percentage of TUJ1 + iNs reprogrammed by miR-124 + ISX9 + siCntl (n = 5 independent experiments) or miR-124 + ISX9 + siTet1 (n = 4 independent experiments) that were also positive for SYN1 and MAP2 on day 11. **E.** Representative images of a miR-124 + ISX9 + siCntl-iN and a miR-124 + ISX9 + siTet1-iN on day 11 processed by the Filament Tracer module in Imaris. Quantification of the maximum primary neurite length per cell (**F**) and average primary neurite length per cell (**G**) in miR-124 + ISX9 + siCntl-iNs (n = 40 cells) and miR-124 + ISX9 + siTet1-iNs (n = 23 cells) via the Filament Tracer module in Imaris. **H.** Number of Sholl intersections relative to the distance from the soma (in μm) (Sholl intersections’ distribution) for miR-124 + ISX9 + siCntl-iNs (n = 40 cells) and miR-124 + ISX9 + siTet1-iNs (n = 23 cells). **I.** Quantification of the number of segment (neurite) branch points per cell for miR-124 + ISX9 + siCntl-iNs (n = 40 cells) and miR-124 + ISX9 + siTet1-iNs (n = 23 cells) via Sholl analysis of the Filament Tracer module in Imaris. The cells that were analyzed via the Filament Tracer module in Imaris were collected from 3 independent experiments for each condition. RT‒qPCR analysis of the mRNA levels of the following transcriptional regulators related to neuronal differentiation: *Baf53b* (**J**), *NeuroD1* (**K**) and *Myt1l* (**L**) in astrocytes reprogrammed with miR-124 + ISX9 -/+ siTet1 on day 7. n = 3 independent experiments *p < 0.05, **p < 0.01, ***p < 0.001

Next, we expanded our study at a later time point, on day 11, focusing on miR-124 + ISX9-iNs, which, according to our previous findings [14], exhibit greater morphological differentiation in comparison to miR-124-iNs. Initially, we co-immunostained cells for TUJ1 and the more mature neuronal markers SYNAPSIN1 (SYN1) and microtubule-associated protein 2 (MAP2) (Fig. 3A). *Tet1* silencing, which was still efficient at this time point as estimated by qRT-PCR (Suppl. Figure 4A), resulted in a significant reduction in the percentage of all TUJ1 + cells by 25.7% (from 72.3% ± 4.4% to 53.8% ± 5.0%) (Fig. 3B) and in the percentage of TUJ1 + cells with a differentiated neuronal morphology by 37.9% (from 74.2% ± 3.0% to 46.1% ± 5.6%) (Fig. 3C). *Tet1* knockdown also conferred a strong reduction in the percentage of the SYN1-MAP2 double-positive TUJ1 + cells by 42.1% (from 92.6% ± 2.4% to 53.6% ± 18.5%) (Fig. 3D). Interestingly, *Tet1* silencing strongly impacted the protein levels of SYN1, as it greatly reduced SYN1 positivity in TUJ1 + cells (Suppl. Figure 4B), while it only slightly decreased MAP2 levels (Suppl. Figure  4 C). Next, we performed a more thorough morphometric analysis to further characterize the effect of *Tet1* silencing on the morphological differentiation of miR-124 + ISX9-iNs. For this purpose, we performed Sholl analysis using the Filament Tracer module in Imaris in miR-124 + ISX9-iNs and miR-124 + ISX9 + siTet1-iNs (representative images are shown in Fig. 3E), which were co-stained for SYN1, MAP2 and TUJ1 (Suppl. Figure 4 D). We estimated the Sholl intersections’ distribution in order to calculate the neurite complexity and measured the length of the primary neurites, along with the total number of primary neurites. Our analysis revealed that miR-124 + ISX9 + siTet1-iNs exhibited shorter processes, as indicated by a significant reduction in both their longest (Fig. 3F) and average primary neurite length (Fig. 3G), which was also evident in the rapid decline in the Sholl intersections’ distribution curve (Fig. 3H). However, their branching complexity did not differ from that of the miR-124 + ISX9-iNs, as indicated by the similar peak in the Sholl intersections’ distribution (Fig. 3H), the number of segments per branch point (Fig. 3I) and the number of primary neurites per cell (Suppl. Figure 4E). The above results point to hindered differentiation of miR-124 + ISX9-iNs due to the decrease in TET1, which seems to impact neurite elongation and synaptic maturation.

To further understand how TET1 contributes to the differentiation of miR-124 + ISX9-iNs, we studied the mRNA levels of transcriptional regulators known to play important roles in the establishment of the neuronal fate during reprogramming, such as the TFs *NeuroD1* and *Myt1l* and the chromatin remodeling factor *Baf53b*, which were previously shown to be uniquely upregulated by ISX9 in our system [14]. qRT‒PCR analysis revealed that silencing *Tet1* led to a significant reduction in *Baf53b* mRNA levels by 78.1% ± 1.0% (Fig. 3J), *NeuroD1* levels by 47.9% ± 4.6% (Fig. 3K) and *Myt1l* levels by 64.3% ± 4.7% (Fig. 3L), pointing to a strong involvement of TET1 in the regulation of crucial players of neurogenic reprogramming. Importantly, we also assessed the effect of *Tet1* silencing on the mRNA levels of *Zfp169* (identified as the top 1 and top 4 TF in miR-124 and miR-124 + ISX9 conditions, respectively), which is included in the TET1 regulon, and verified its significant downregulation by 51.3% ± 6.5% in the absence of *Tet1* (Suppl. Figure 4 F).

### TET1 Regulates Genes Involved in Neuron Projection Morphogenesis and Synaptic Signaling in Differentiating miR-124 + ISX9-iNs

**Fig. 4:**
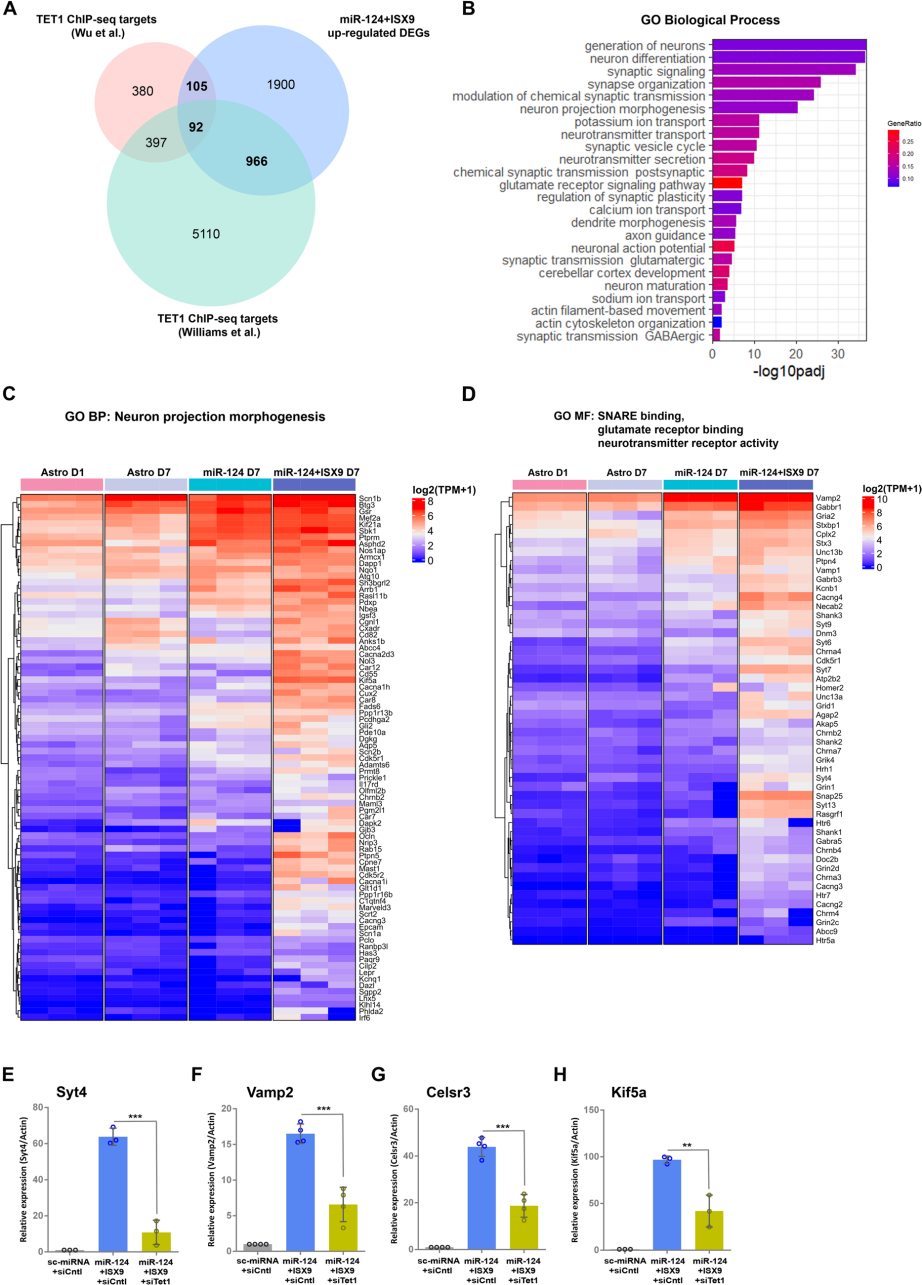
TET1 regulates genes involved in neuron projection morphogenesis and synaptic signaling in differentiating miR-124 + ISX9-iNs. **A.** Venn diagram representing the overlap between TET1 direct targets identified in two independent TET1-ChIP experiments and genes upregulated in miR-124 + ISX9-iNs compared to astrocytes on day 1 (log_2_(fold change) > 1, padj < 0.01). **B.** Gene Ontology (GO) Biological Processes (BP) terms enriched for the 1,163 TET1 direct targets upregulated in miR-124 + ISX9-iNs on day 7. The GO terms are ranked by p adjusted values and color coded by the gene ratio (the number of enriched genes divided by the total number of genes in the GO term). Heatmaps showing the expression of genes associated with the GO BP terms “neuron projection morphogenesis” (**C**) and the GO MF terms “SNARE binding, glutamate receptor binding and neurotransmitter binding activity” in astrocytes and iNs (**D**). RT‒qPCR analysis of the mRNA levels of genes related to synaptic activity: *Syt4* (n = 3 independent experiments) *(E*), *Vamp2* (n = 4 independent experiments) (**F**), *Celsr3* (n = 4 independent experiments) (**G**) and *Kif5a* (n = 3 independent experiments) (**H**) in astrocytes reprogrammed with miR-124 + ISX9 -/+ siTet1 on day 7. **p < 0.01, ***p < 0.001

To gain more thorough insight into the role of TET1 in miR-124 + ISX9-iNs’ differentiation, we sought to identify direct targets of TET1 that are differentially expressed in miR-124 + ISX9-iNs. To this end, we used publicly available TET1-ChIP-seq data from two studies [61, 62] and filtered TET1 direct targets combined from both studies with the DEGs that are upregulated in miR-124 + ISX9-iNs (log_2_FC > 1, padj < 0.01). We identified 1,163 genes (Fig. 4A), which were further subjected to Gene Ontology (GO) enrichment analysis using g profiler. The top GO Biological Processes (BP) terms inferred from this analysis included generation of neurons, neuron differentiation, synaptic signaling, neuron projection morphogenesis, potassium ion transport and neurotransmitter transport (Fig. 4B). Furthermore, among the top enriched GO Molecular Function (MF) terms were voltage- and ligand-gated cation channels, mainly potassium channels, as well as cytoskeletal binding proteins and GTPase activators, neurotransmitter receptors, primarily glutamate, and components of the SNARE complex (Suppl. Figure  5A), whereas GO Cellular Component (CC) term analysis revealed the synapse (mostly glutamatergic) and the neuron projection as the top enriched cell compartments (Suppl. Figure 5 B). Genes from selected major GO terms are presented in heatmaps, i.e., GO BP neuron projection morphogenesis (Fig. 4C), GO MF SNARE binding and neurotransmitter receptor signaling (Fig. 4D) and GO BP synaptic signaling and synaptic plasticity (Suppl. Figure  5C and D). On the basis of these data, we selected genes involved in the aforementioned biological processes for further validation, namely, Synaptotagmin 4, *Syt4*, the Vesicle-associated membrane protein 2, *Vamp2*, with role in synaptic vesicle trafficking, the Cadherin EGF LAG Seven-Pass G-Type Receptor 3, *Celsr3*, with role in axonal navigation and neuronal migration and the Kinesin family member 5 A, *Kif5a,* implicated in neurite outgrowth. Our RT‒qPCR analysis on day 7 verified the strong impact of *Tet1* silencing on the mRNA levels of all the genes that were studied, with *Tet1* silencing reducing the expression of *Syt4* by 82.6% ± 9.9% (Fig. 4E), *Vamp2* by 60.2% ± 14.7% (Fig. 4F), *Celsr3* by 57.4% ± 10.2% (Fig. 4G) and *Kif5a* by 56.6% ± 17.7% (Fig. 4H).

### Silencing *Lin28a* Affects the Morphological Neurogenic Transition of miR-124 + ISX9-iNs

TET1 has been reported to be recruited by TFs to specific gene loci to regulate gene transcription [62–64]. LIN28A, which is the top 2 TF of the miR-124 + ISX9 condition and also uniquely regulated by ISX9, has been reported to bind to DNA in active promoters, sequestering and forming complexes with TET1 to regulate DNA methylation and gene expression of specific genes in ESCs [63]. Prompted by these findings, which link TET1 to LIN28A in the co-regulation of specific genes, we next investigated the possible collaboration of the two transcriptional regulators in the control of genes upregulated by ISX9 in our system.

**Fig. 5:**
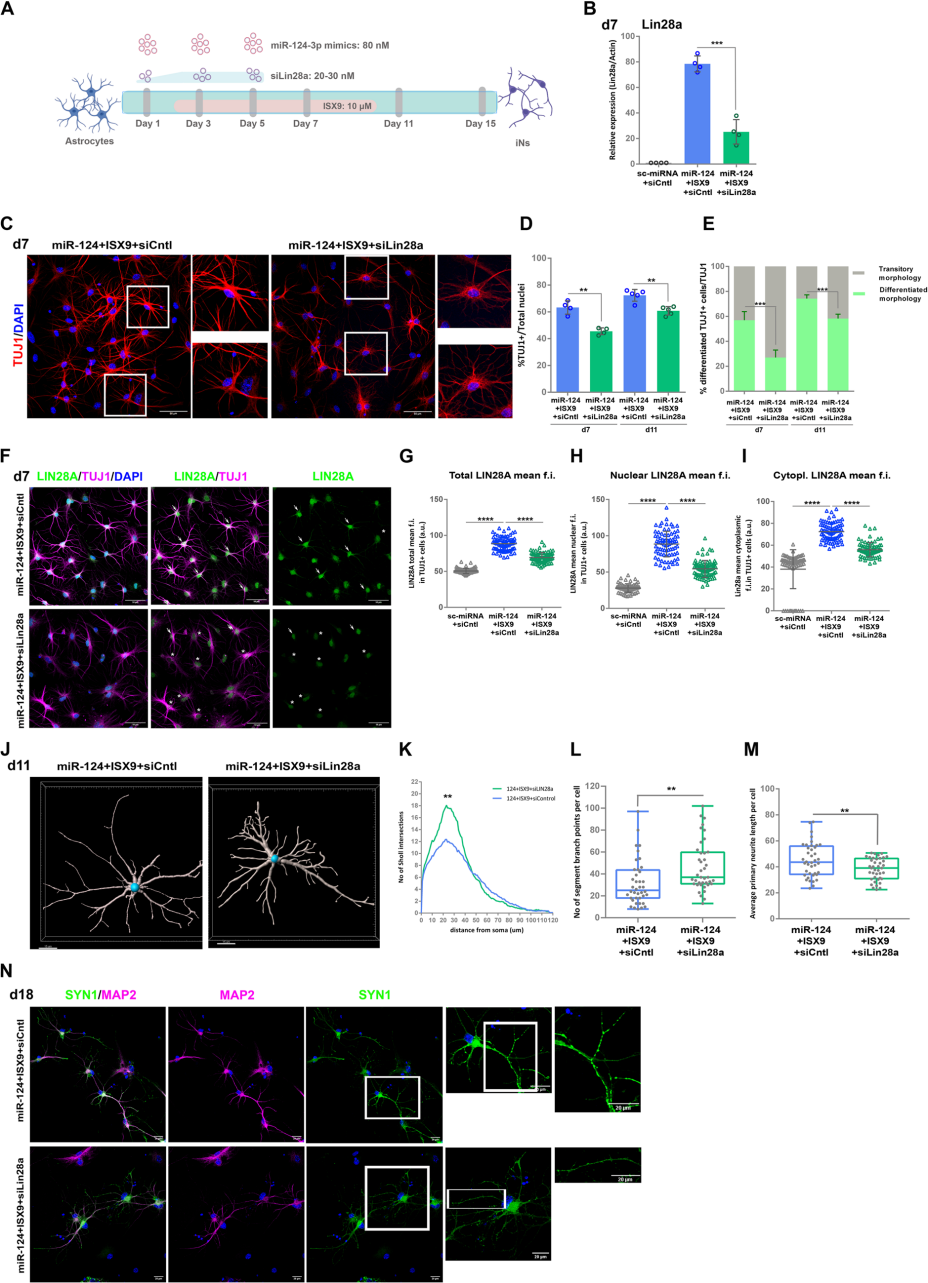
Silencing of *Lin28a* affects the morphological transition of miR-124 + ISX9-iNs. **A.** Schematic representation of the protocol used to silence *Lin28a* during the reprogramming process. **B.** Estimation of the degree of *Lin28a* mRNA silencing on day 7 of reprogramming by miR-124 + ISX9 -/+ siLin28a (n = 4 independent experiments) by qRT‒PCR. **C.** Immunostaining of astrocytes reprogrammed with miR-124 + ISX9 -/+ siLin28a on day 7 with an anti-TUJ1 antibody (in red); the inset areas show representative cell morphologies. **D.** Quantification of the percentage of TUJ1 + reprogrammed cells by miR-124 + ISX9 -/+ siLin28a on day 7 (n = 4 independent experiments) and on day 11 (n = 5 independent experiments). **E.** Presentation of the proportion of differentiated TUJ1 + iNs (green portions of the bars) in the total TUJ1 + population (as quantified in **D** for each condition and set to 100%) (the gray portions of the bars indicate the proportion of TUJ1 + iNs exhibiting a transitory still not differentiated morphology). **F.** Immunostaining of astrocytes reprogrammed with miR-124 + ISX9 -/+ siLin28a on day 7 with an anti-TUJ1 antibody (in magenta) and an anti-LIN28A antibody (in green); representative cells exhibiting high LIN28A levels and a differentiated morphology (categories 1 or 2) are indicated with arrows, while representative cells with low LIN28A levels and a less differentiated morphology (categories 2 or 3) are indicated with asterisks. Measurement of the total mean fluorescence intensity (f.i.) (**G**), nuclear mean f.i. (**H**) and cytoplasmic mean f.i. (**I**) of LIN28A protein levels on day 7 in control cells treated with sc-miRNA + siCntl and TUJ1 + reprogrammed cells treated with miR-124 + ISX9 -/+ siLin28a (n = 80 cells for each condition from 3 independent experiments) (mean ± SEM, p < 0.0001, two tailed t-test assuming unequal variance). **J.** Representative images of a miR-124 + ISX9 + siCntrl-iN and a miR-124 + ISX9 + siLin28a-iN on day 11 processed by the Filament Tracer module in Imaris. **K.** Representation of the number of Sholl intersections relative to the distance from the soma (in μm) (Sholl intersections’ distribution) for miR-124 + ISX9 + siCntrl-iNs (n = 40 cells) and miR-124 + ISX9 + siLin28a-iNs (n = 39 cells). **L.** Quantification of the number of segment (neurite) branch points per cell for miR-124 + ISX9 + siCntrl-iNs (n = 40 cells) and miR-124 + ISX9 + siLin28a-iNs (n = 39 cells) using Sholl analysis with the Filament Tracer module in Imaris. **M.** Quantification of the average primary neurite length per cell in miR-124 + ISX9 + siCntrl-iNs (n = 40 cells) and miR-124 + ISX9 + siLin28a-iNs (n = 39 cells) using the Filament Tracer module in Imaris. The cells that were analyzed with the Filament Tracer module in Imaris were collected from 3 independent experiments for each condition. *p < 0.05, **p < 0.01, ***p < 0.001. **N.** Coimmunostaining of miR-124 + ISX9 + siCntl-iNs and miR-124 + ISX9 + siLin28a-iNs on day 18 with an anti-MAP2 antibody (in magenta) and an anti-SYN1 antibody (in green); the inset areas show higher magnifications of representative cells and their processes stained with SYN1

To this end, we were initially interested in investigating the contribution of LIN28A in the reprogramming and neuronal differentiation of miR-124 + ISX9-iNs instructed by ISX9. For this purpose, we knocked down *Lin28a* using a pool of 4 siRNAs specific for mouse *Lin28a* following the protocol presented in Fig. 5A. Since *Lin28a* is uniquely upregulated by ISX9, we limited this study to the miR-124 + ISX9 condition and focused on later time points, days 7–18, to study iNs’ differentiation. Knocking down *Lin28a* with siLin28a resulted in a 73.6% ± 8.5% reduction in *Lin28a* mRNA levels, as estimated by qRT‒PCR (Fig. 5B). Notably, the silencing of *Lin28a* did not significantly affect the mRNA levels of its paralog gene *Lin28b;* however, a slight reduction of 23.8% ± 4.5% was observed (Suppl. Figure  6A). Next, we assessed the impact of *Lin28a* silencing on the reprogramming efficiency and differentiation of miR-124 + ISX9-iNs via the same strategy used for siTet1 to estimate the number of TUJ1 + cells and the degree of their morphological differentiation. Our analysis revealed that the silencing of *Lin28a* led to a small but significant reduction in TUJ1 + cells by 23.9% (from 60.0% ± 8.5% to 45.6% ± 5.5%) on day 7 and by 14.5% (from 72.3% ± 4.4% to 61.9% ± 3.2%) on day 11 of reprogramming (Fig. 5C and D, insets in Fig. 5C show representative morphologies). Most importantly, the silencing of *Lin28a* significantly affected the morphological differentiation of miR-124 + ISX9-iNs, as it decreased the percentage of TUJ1 + miR-124 + ISX9-iNs with a differentiated morphology by 52.1% (from 56.9% ± 6.9% to 27.3% ± 6.3%) on day 7 and 21.6% (from 74.2% ± 3.0% to 58.2% ± 3.5%) on day 11 (Fig. 5C and E). Of note, measuring the mean fluorescence intensity (f.i.) of LIN28A protein both in the nucleus and the cytoplasm of miR-124 + ISX9 reprogrammed TUJ1 + cells on day 7, we verified the reduction of LIN28A at the protein level and correlated the low LIN28A levels with the impaired differentiation of TUJ1 + cells that undergo reprogramming (Fig. 5F-I and Suppl. Figure 6B). Furthermore, staining of iNs for TUJ1 along with the more mature neuronal markers SYN1 and MAP2 on day 11 indicated that almost all TUJ1 + miR-124 + ISX9-iNs and miR-124 + ISX9-siLin28a-iNs were also positive for SYN1 and MAP2 (Suppl. Figure  6 C and D). Intriguingly, miR-124 + ISX9 + siLin28a-iNs exhibited a characteristic multipolar and hyper-branched morphology on day 11, in contrast to the morphology of miR-124 + ISX9-iNs, which typically presented a more differentiated iN appearance with fewer and longer neurites (Suppl. Figure 5C). To further characterize this aberrant morphology of iNs following *Lin28a* knockdown, we performed the same morphometric analysis as in Fig. 3 using the Filament Tracer module in Imaris (representative images from this analysis are shown in Fig. 5J and Suppl. Figure 6E). Indeed, the Sholl intersections’ distribution showed that miR-124 + ISX9 + siLin28a-iNs presented a significant increase in the number of intersections per cell (Fig. 5K), along with a significant increase in the number of segment branch points per cell (Fig. 5L). These observations, along with the finding that miR-124 + ISX9 + siLin28a-iNs harbor more primary neurites (Suppl. Figure 6F), verify the evident increase in multipolarity and primary neurite branching due to the decrease in *Lin28a*. Furthermore, we observed a significant decrease in the average (Fig. 5M) and maximum primary neurite length (Suppl. Figure 6G) of miR-124 + ISX9 + siLin28a-iNs, implying that a hindrance in neurite elongation after the silencing of *Lin28a* impacted iNs’ differentiation. Similarly, labeling of iNs in longer time points with SYN1, namely on day 15 and day 18, revealed that, in contrast to miR-124 + ISX9-iNs, miR-124 + ISX9 + siLin28a-iNs did not exhibit punctate expression of SYN1 along their neurites (Suppl. Figure  7 A and Fig. 5N) a finding that is also supported by the staining pattern of another synaptic protein, SYP (Synaptophysin) (Suppl. Figure 7B).

Collectively, both molecular phenotype and morphometric analysis revealed that silencing *Lin28a* moderately hampers the neurogenic conversion of miR-124 + ISX9-iNs; however, it evidently affects their morphological progression toward a more differentiated neuronal phenotype. Along this line, we observed that silencing *Lin28a* significantly reduced the mRNA levels of *NeuroD1* by 64.2% ± 10.1% on day 7 (Suppl. Figure  7 C), which plays an important role in iNs’ differentiation. Interestingly, we also identified the microtubule binding protein DCX, a marker of neuroblasts, as being significantly downregulated by the knockdown of *Lin28a* by 58.4% ± 26.0% (Suppl. Figure 7D), thus linking LIN28A to the regulation of microtubule dynamics and cytoskeletal rearrangements that are crucial during neurogenesis and neuronal differentiation. On the other hand, *Lin28a* silencing did not significantly affect the mRNA levels of *Baf53b* (Suppl. Figure 7E), but it resulted in moderate but not significant downregulation of *Myt1l* mRNA levels (Suppl. Figure  7 F). Finally, we assessed the effect of *Lin28a* silencing on the mRNA levels of the TFs *Tshz2* (top 1 in miR-124 + ISX9 condition) and *Myt1* (top 32 in miR-124 + ISX9 condition and uniquely upregulated by ISX9), which were identified as part of the LIN28A regulon (Suppl. File 3), and verified their significant downregulation by 47.0% ± 4.2% (Suppl. Figure 7G) and 37.1% ± 2.5% (Suppl. Figure 7H), respectively, after *Lin28a* silencing.

### LIN28A and TET1 Co-Regulate a Set of Synaptic Genes Expressed in Differentiating miR-124 + ISX9-iNs

**Fig. 6:**
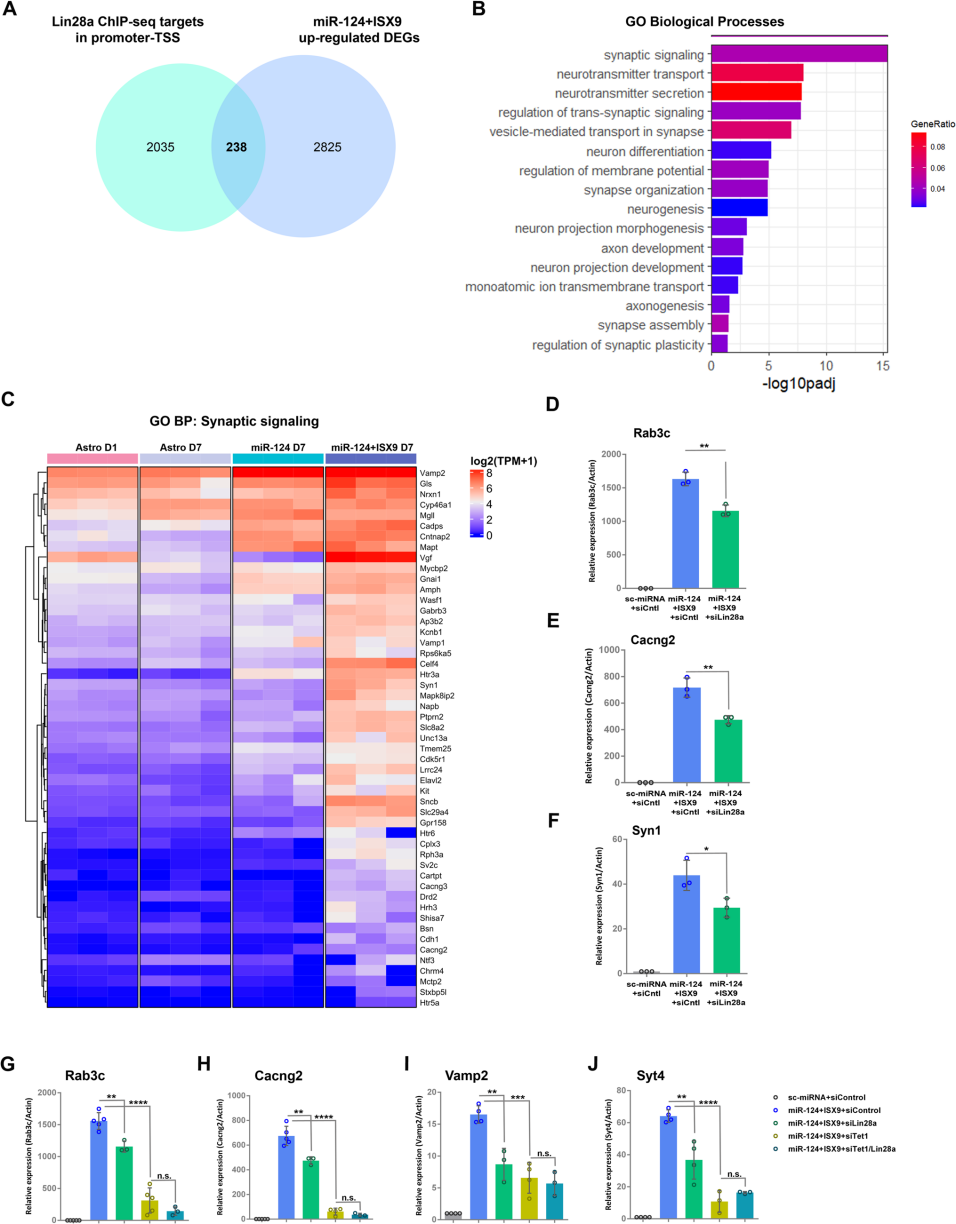
LIN28A and TET1 co-regulate a set of synaptic genes expressed in differentiating miR-124 + ISX9-iNs. **A.** Venn diagram representing the overlap between LIN28A direct targets derived from a publicly available LIN28A-ChIP experiment and genes upregulated in miR-124 + ISX9-iNs compared to astrocytes on day 1 (log_2_(fold change) > 1, padj < 0.01). **B.** Gene Ontology (GO) Biological Processes (BP) terms enriched for the 238 LIN28A direct targets upregulated in miR-124 + ISX9-iNs. The GO terms are ranked by p adjusted values and color coded by the gene ratio (the number of enriched genes divided by the total number of genes in the GO term). **C.** Heatmap showing the expression of genes associated with the GO BP “synaptic signaling”. RT‒qPCR analysis of the mRNA levels of genes related to synaptic activity, *Rab3c *(**D**), *Cacng2* (**E**) and *Syn1* (**F**), in astrocytes reprogrammed with miR-124 + ISX9 -/+ siLin28a on day 7. n = 3 independent experiments. RT‒qPCR analysis of the mRNA levels of the genes *Rab3c *(**G**), *Cacng2* (**H**), *Vamp2* (**I**) and *Syt4* (**J**) in astrocytes reprogrammed with miR-124 + ISX9 + siControl (n = 5 independent experiments for *Rab3c* and *Cacng2* and n = 4 independent experiments for *Vamp2* and *Syt4*), miR-124 + ISX9 + siLin28a (n = 3 independent experiments for *Rab3c*, *Cacng2* and *Vamp2* and n = 4 independent experiments for *Syt4*), miR-124 + ISX9 + siTet1 (n = 5 independent experiments for *Rab3c*, n = 4 independent experiments for *Cacng2* and *Vamp2 and* n = 3 independent experiments for *Syt4)* or miR-124 + ISX9 + siTet1/Lin28a (n = 3 independent experiments) on day 7. *p < 0.05, **p < 0.01, ***p < 0.001, ****p < 0.0001

Next, we aimed to further understand the contribution of LIN28A to the differentiation of iNs, focusing on its reported role in DNA binding. Therefore, we sought to identify direct targets of LIN28A that are differentially expressed in miR-124 + ISX9-iNs. For this purpose, we used publicly available LIN28A ChIP-seq data [63] and focused only on genes that were found to be bound by LIN28A at their transcription start site (TSS) or their proximal promoter. We next filtered this list of genes with the upregulated DEGs in miR-124 + ISX9-iNs (log_2_FC > 1, padj < 0.01) and identified 238 common genes (Fig. 6A), which were further analyzed with g profiler. The top enriched GO BP terms included synaptic signaling, neurotransmitter transport and secretion, neuron differentiation, regulation of membrane potential and neuron projection morphogenesis (Fig. 6B). Additionally, the synapse and neuron projection were the top enriched GO CC terms, whereas the synaptic vesicle membrane, excitatory synapse and SNARE complex presented high gene ratios (Suppl. Figure  8 A). Genes from selected GO terms are presented in heatmaps, such as GO BP synaptic signaling (Fig. 6C) and GO BP neuron projection morphogenesis (Suppl. Figure 8B). On the basis of these findings, we studied the impact of *Lin28a* silencing on the mRNA levels of selected genes involved in the abovementioned biological processes, namely, the Ras-related small GTPase, *Rab3c*, with role in synaptic vesicle trafficking, the Calcium Voltage-Gated Channel Auxiliary Subunit Gamma 2, *Cacng2*, with role in AMPA receptor trafficking and channel gating, also implicated in synaptic plasticity and Synapsin1, *Syn1*, with role in synaptic vesicle trafficking and neurotransmitter transport. Our qRT‒PCR analysis on day 7 revealed that the silencing of *Lin28a* induced a moderate but significant reduction in *Rab3c* mRNA levels by 25.0% ± 3.8% (Fig. 6D), *Cacng2* levels by 30.2 ± 12.7% (Fig. 6E) and *Syn1* levels by 32.7% ± 6.6% (Fig. 6F), indicating the implication of LIN28A in the regulation of genes related to synaptic vesicle trafficking and other synaptic functions.

Next, we sought to identify genes that are both direct targets of TET1 and LIN28A to investigate the possible synergistic action between these two factors in our system. For this purpose, we compared the direct targets of TET1 and LIN28A that were identified as upregulated in miR-124 + ISX9-iNs and identified 146 common genes (Suppl. Figure  8 C), including many of those that were already presented here, such as *Rab3c*, *Cacng2*, *Syn1* (as LIN28A direct targets) and *Vamp2* (as TET1 direct target). We further established a protocol for the cosilencing of *Tet1* and *Lin28a* (Suppl. Figure 8D) and studied the impact of *Lin28a* and *Tet1* silencing alone, as well as in combination, via qRT‒PCR. Our analysis revealed that *Rab3c*, *Cacng2* and *Syn1* were strongly reduced by *Tet1* silencing by 79.6% ± 12.5% for *Rab3c* (Fig. 6G), 90.3% ± 4.6% for *Cacng2* (Fig. 6H) and 56.3% ± 12.4% for *Syn1* (Suppl. Figure 8E*)*, whereas co-silencing of *Tet1* and *Lin28a* did not result in a further significant reduction. On the other hand, the silencing of *Lin28a* significantly reduced the mRNA levels of *Vamp2* by 45.5% ± 16.1%, whereas similar to the other genes studied here, the parallel silencing of *Tet1* and *Lin28a* did not induce further significant downregulation of *Vamp2* relative to the effect of *Tet1* (Fig. 6I). Interestingly, we observed a similar pattern of regulation for the TET1 direct target *Syt4* (Fig. 6J), which was not identified as a direct target of LIN28A. Additionally, we also identified *NeuroD1* as a gene that is significantly and robustly affected both by the silencing of *Tet1* and *Lin28a* (Suppl. Figure  8 F); however, NeuroD1 was not identified as a direct gene of either TET1 or LIN28A in our analysis.

Overall, we highlighted a set of genes with synaptic roles, which were previously identified via ChIP-seq experiments as direct targets of TET1 and LIN28A, that are coregulated by TET1 and LIN28A in miR-124 + ISX9-iNs, with TET1 being the major regulator and LIN28A acting primarily to enhance the TET1 effect. These observations could imply that the upregulation of *Lin28a* by ISX9 provides a novel partner for TET1, reinforcing its action at neuron-specific gene loci. Additionally, we identified neuronal differentiation genes that are coregulated by TET1 and LIN28A, possibly at different levels of regulation, such as the post-transcriptional level, suggesting that these two factors converge on the regulation of neuron-specific genes.

## Discussion

In this study, we revealed the transcriptional regulatory network that is established by miR-124 upon its overexpression in mouse primary astrocytes and identified the impact of the neurogenic compound ISX9 on the reinforcement of part of this network, as well as on the activation of novel neurogenic regulators. Our analysis of the top transcriptional regulators revealed that miR-124 is essential for the establishment of a core regulatory network, upon which ISX9 further acts to exert its neurogenic action. Following an unbiased approach, we identified the DNA demethylase TET1 as a top transcriptional regulator of the astrocytic fate switch toward the neuronal identity, which is instructed by miR-124 and further enhanced by ISX9. Our *Tet1* silencing experiments strongly indicate that TET1 plays an important role in miR-124-induced early reprogramming and thus reveal the central role of this epigenetic action of miR-124 on the astrocytic DNA methylome during the early stages of reprogramming. Our findings are in accordance with previous studies reporting that the combination of miR-124 with miR-9/9* strongly affects the chromatin dynamics and DNA methylation of human fibroblasts undergoing neurogenic reprogramming [22, 26]. Importantly, TET family proteins, mostly TET1, have also been shown to play critical roles in the epigenetic conversion of human fibroblasts to induced dopaminergic neurons (iDAs) [65]. DNA demethylation by TET1 has been reported to be critical both in embryonic [50] and adult hippocampal neurogenesis [58, 59]. Interestingly, TET1 has been reported to control the demethylation of miR-124 promoter, regulating miR-124 abundance during adult hippocampal neurogenesis [59]. This TET1-mediated regulation of miR-124, along with our observations that miR-124 overexpression employs TET1 for its reprogramming action, suggests a positive feedback mechanism that enhances the neurogenic action of these two factors. Consequently, our results unravel a novel key role for TET1 in the regulation of the astrocytic fate conversion toward the neuronal identity by miR-124.

Interestingly, our data indicate that TET1 is also instrumental for the ISX9-mediated reinforcement of iNs’ differentiation. Indeed, we show that the silencing of *Tet1* affects not only early neuronal conversion, but also the expression levels of ISX9-induced neuronal differentiation genes and the morphological differentiation of miR-124 + ISX9-iNs. In addition to its role in neurogenesis, TET1 has been shown to be pivotal for the DNA demethylation required for activity-dependent neuronal gene expression in the adult brain [66]. Importantly, a recent study identified the TF EGR1, which plays important role in brain development, learning and long-term neuronal plasticity [67], as a recruiter of TET1 to EGR1 binding sites, enabling locus-specific epigenetic regulation upon neuronal activation in the postnatal mouse frontal cortex [64]. EGR1 activity was found to be moderately increased by ISX9 in our analysis (Suppl. File 1); however, it was not present in the regulatory network established at the rather early time point of day 7. Furthermore, TET1 has also been reported to be sequestered by LIN28A in active promoters to facilitate gene expression in ESCs [63]. The aforementioned study reported for the first time the DNA-binding activity of LIN28A, in addition to its well-known action as an RNA-binding protein with a prominent role in inhibiting let-7 miRNA maturation [68]. The appearance of LIN28A as the top 2 transcriptional regulator in miR-124 + ISX9 condition, as well as its unique upregulation by ISX9, prompted us to investigate its role in ISX9-induced iN differentiation and, further, its possible collaboration with TET1. *Lin28a* silencing moderately contributed to the neuronal conversion of miR-124 + ISX9-iNs; however, it highlighted the stronger involvement of LIN28A in the morphological transition of iNs toward a more differentiated neuronal phenotype. Our findings suggest a role of LIN28A in the control of cytoskeletal reorganization and neurite outgrowth, which is in agreement with recent studies reporting the involvement of LIN28A in neurite outgrowth in primary cortical neurons [69]. Importantly, the hyper-branched phenotype observed in miR-124 + ISX9-iNs following *Lin28a* silencing resembles the extensive neurite branching reported in morphologically defective DCX-KO iPSC-derived neurons [70] and mouse Dcx-KO neurons [71]. These findings are in accordance with our observation that the silencing of *Lin28a* robustly downregulates *Dcx* mRNA levels, a finding that requires further investigation. Furthermore, LIN28A has well-established roles in cortical neurogenesis [72, 73], as well as in adult hippocampal neurogenesis, controlling the proliferation of NPCs and the development of newborn neurons in response to Wnt signaling [52]. Interestingly, ISX9 has been recently reported to act as an agonist of the Wnt/β-catenin pathway [74], indicating that LIN28A might be upregulated by the Wnt pathway in our system, a mechanism that remains to be elucidated in subsequent studies. Collectively, our findings support, for the first time, a role for LIN28A in the neurogenic conversion of astrocytes through the action of ISX9. Notably, LIN28A has emerged as a survival enhancer of grafted NSCs in a Parkinson’s disease rat model [75], which is in line with our in vivo findings regarding the ISX9-reinforced iN survival in the injured mouse cortex [14].

Our gene expression analysis of TET1 ChIP-seq and LIN28A ChIP-seq common targets participating in mechanisms of synaptic activity points to a combined action of TET1 and LIN28A in the regulation of their expression levels. More specifically, these results reveal a possible mechanism through which ISX9 promotes further differentiation of iNs by upregulating LIN28A, which, among other functions, acts as a novel partner of TET1, facilitating further upregulation and expansion of neuron-specific target genes' expression. However, our experimental approach cannot distinguish between the DNA- and RNA-binding action of LIN28A. It is therefore possible that our observations stem from either the collaboration of TET1 and LIN28A at specific genetic loci or alternatively from their combined action at the DNA demethylation level for TET1 and the mRNA level [76] or the inhibition of let-7 miRNA for LIN28A [68, 77]. It is therefore interesting to further explore the possibility that LIN28A could act as a new partner for TET1 in differentiating neurons, as it has been shown in ESCs. A limitation of our approach is that the direct TET1 and LIN28A targets were derived from ChIP-seq experiments performed in ESCs, a cell system that differs from astrocytes and immature neurons, thus the identified set of genes may be an under-representation of the true TET1 and LIN28A direct targets in our cell system.

Collectively, the present study uncovered novel transcriptional regulators with significant roles in controlling the transcriptional changes imposed by miR-124 and ISX9 in astrocytes during their neurogenic conversion. TET1 has emerged as a crucial regulator of the early neurogenic switch of astrocytes by miR-124, as well as a central regulator of the differentiation of iNs in collaboration with novel neurogenic factors upregulated by ISX9, such as LIN28A. Thus, we revealed DNA demethylation as an important aspect of the reprogramming action of miR-124, that is reinforced by the neurogenic activity of ISX9. Importantly, this study emphasizes the candidacy of TET1 as a novel regulator of neurogenic reprogramming that could be added in an improved miR-124/ISX9 in vivo reprogramming cocktail to enhance their combined neurogenic action following brain injury and neurodegeneration, a hypothesis that needs further exploration in a future study.

## Methods

### Primary Cultures of Postnatal Cortical Astrocytes

Primary postnatal astrocyte cultures from P3‒P5 mice were prepared as follows. Cerebral cortices from 2‒3 P3‒P5 C57BL/6 mice were collected in ice-cold HBSS (Invitrogen), and the tissue was washed three times with HBSS and digested with 0.04% trypsin (Sigma) and 10 μg/ml DNAse (Sigma) for 5 min at 37 °C. After digestion, the cells were mechanically dissociated, centrifuged for 5 min at 850 rpm (120 × g), resuspended in DMEM (4.5 g/L glucose; Invitrogen) supplemented with 10% FBS (Invitrogen) and 1% penicillin/streptomycin (Pen/Strep) (Sigma) and placed in a T75 flask precoated with poly-D-lysine (PDL) (Sigma). When the culture reached confluence (usually after 7 days), the flask was shaken in a horizontal shaker at 200–250 rpm for 20 h to obtain a pure astrocytic culture that was free of neurons, oligodendrocytes and microglia. The remaining cells were digested with 0.5% trypsin–EDTA (Invitrogen) for 5 min at 37 °C, centrifuged at 850 rpm, resuspended in fresh DMEM containing 4.5 g/L glucose, 10% FBS, and 1% Pen/Strep and divided into two new T75 flasks precoated with PDL. Half of the medium was changed every two days.

### In vitro reprogramming protocol

For the reprogramming of astrocytes to induced neurons, 40,000 astrocytes were seeded on 10 mm coverslips coated with 20 μg/ml poly-L-ornithine (PLO) (Sigma) overnight and 5 μg/ml laminin for 3 h at 37 °C (Sigma). Once the cells reached > 90% confluence (usually after 1–2 days), they were transfected with 80 nM miR-124-3p mimics, scrambled (sc-miRNA) mimics (negative control) or miRNA-Dy546 mimics (transfection control) (Thermo) via Lipofectamine 2000 (Invitrogen) according to the manufacturer’s instructions (day 1). The next day, the astrocytic medium (DMEM containing 4.5 g/L glucose, 10% FBS, and 1% Pen/Strep) was replaced with the following reprogramming medium: Neurobasal medium (Invitrogen) supplemented with 1X B-27 (Invitrogen), 1X GlutaMAX (Invitrogen) and 200 mM ascorbic acid (Sigma). The same process of transfection was repeated twice on days 3 and 5. On day 7, the reprogramming medium was changed to the following neuronal differentiation medium: Neurobasal medium supplemented with 1X B-27, 1X GlutaMAX, 20 ng/ml BDNF (R&D Systems), 0.5 mM cAMP (Sigma) and 200 mM ascorbic acid. In the miR-124 + ISX9-reprogrammed cells, 10 μΜ ISX9 chemical compound (MedChemExpress) was added from days 2–10. All the media added to the reprogrammed cells were preconditioned for 24 h in a confluent astrocytic culture.

### Silencing of *Tet1* and *Lin28a* Using a Pool of siRNAs

For the silencing of mouse *Tet1*, astrocytes were transfected with 20 nM siTet1 (a pool of 4 siRNAs, Dharmacon) or control siRNA (siCntl) (Dharmacon) on days 1, 3 and 5 in a mixture of 80 nM miR-124-3p mimics via Lipofectamine 2000 (Invitrogen) according to the manufacturer’s instructions. Accordingly, for the silencing of *Lin28a*, astrocytes were transfected with 20 nM siLin28a (a pool of 4 siRNAs, Dharmacon) or control siRNA (siCntl) (Dharmacon) at day 1 and 30 nM at days 3 and 5 in a mixture with 80 nM miR-124-3p mimics via Lipofectamine 2000. For the long term experiment of 18 days a fourth transfection was performed at day 8 with 30 nM of siLin28a to enhance the observed phenotype by *Lin28a* silencing. For the combined silencing of *Tet1* and *Lin28a*, a different protocol was followed, with separate transfections with siTet1 or siLin28a to ensure adequate downregulation of both genes. For this purpose, on days 1, 3 and 5, astrocytes were transfected with 20 nM siTet1 in a mixture of 80 nM miR-124-3p mimics, whereas on day 2, they were transfected with only 20 nM siLin28a, and on days 4 and 6, they were transfected with 30 nM siLin28a.

### RT‒qPCR Analysis

For the RT‒qPCR analysis experiments, total RNA was extracted using the Nucleospin miRNA Kit (Macherey–Nagel), and 500–800 ng of total RNA was used for cDNA synthesis with Superscript II reverse transcriptase (Invitrogen) according to the manufacturer’s instructions. Quantitative real-time PCR was performed using SYBR Select Master Mix (Applied Biosystems), and samples were run on the StepOnePlus System (Applied Biosystems). The primers used are listed in Table S1. Each sample was analyzed in triplicate, gene expression was calculated via the ΔΔCt method, and all the results were normalized to β-actin expression. Relative expression was estimated by setting the values of sc-miRNA + siCntl-treated astrocytes to 1. All the experiments were performed with at least 3 biological replicates.

### Bioinformatic Analysis

#### Alignment of Fastq Reads with Kallisto and DESeq2 Differential expression Analysis

We used the pseudoaligner tool Kallisto (version 0.41.1) to align all fastq files available at the European Nucleotide Archive under study accession ENA: PRJEB38603, https://www.ebi.ac.uk/ena/browser/view/PRJEB38603. The reference used to construct the Kallisto index was GRCh39 cDNA release 106 (http://ftp.ensembl.org/pub/release-106/fasta/mus_musculus/). To identify differentially expressed genes (DEGs), we used the R library DESeq2 (https://doi.org/10.1186/s13059-014–0550-8) with gene expression at the transcription level, as previously described [78]. Kallisto output was imported into the R (version 4.0.5) programming environment, and a DESeq2 object was created and filtered on the basis of the sum of rows with counts greater than 10. Next, the DESeq function was used to filter results on the basis of a model via the negative binomial distribution and correction method FDR (False Discovery Rate). Genes were considered differentially expressed only if the FDR was < 0.01 and the absolute log_2_-fold change (|log_2_FC|) was > 1.0.

### Regulatory Transcription Network (RTN) Analysis

The RTN package is designed for the reconstruction of transcriptional regulatory networks (TRNs) consisting of a collection of regulated target genes and transcriptional regulators. The package tests the associations between a given transcriptional regulator and all potential targets using RNA-seq data [31, 32]. Transcriptional Network Inference (TNI) was performed via the function tni.constructo() to create a TNI-class object. To that end, we used the normalized matrix of gene expression (from DESeq2 analysis), sample annotation and a list of 1,374 mouse transcription factors on the basis of the curated list of human transcription factors (https://cgs.csail.mit.edu/ReprogrammingRecovery/mouse_tf_list.html). After the construction of the TNI-class object, we computed the mutual information (MI) between a regulator and all potential targets and removed nonsignificant associations via permutation analysis. We subsequently removed unstable interactions by bootstrapping and applied the ARACNe algorithm to remove the weakest interaction in any triplet formed by two transcriptional regulators and a common target gene, preserving the dominant regulator‒target pair. Graphical representations of TRNs were generated via Cytoscape (PubMed ID: 14,597,658). The betweenness centrality (the number of shortest paths between other nodes that run through the node of interest [55, 56] of the TRN) was calculated using the package igraph 2.1.4 https://igraph.org. A node is considered central (high betweenness centrality) if it appears on many shortest paths that connect pairs of nodes. TRNs for ASCL1-iNs and NEUROG2-iNs were generated using publicly available data [57]. Common regulons identified between ASCL1-iNs and miR-124 + ISX9-iNs RTNs or between NEUROG2-iNs and miR-124 + ISX9-iNs RTNs were curated manually.

### Inference of TET1 and LIN28A Direct Targets Using Publicly Available Data

To identify direct targets of TET1, we used publicly available data from two studies in which TET1-ChIP-seq experiments were performed in ESCs [61, 62]. We combined in one list the TET1 target genes whose peaks were found ± 1 kb from their TSS and were considered significant (FDR < 0.01) in the peak detection analysis; these genes were identified by ChIP-seq using two different TET1 antibodies (one binding to the N-terminus and one binding to the C-terminus of TET1 protein) [62]. We also used the genes that were identified as DEGs in Tet1 KO vs WT ESCs and reported to also be bound to TET1 [61]. The TET1 direct target genes inferred from these studies were merged and filtered for genes whose expression was upregulated in miR-124 + ISX9-iNs day 7 vs. astro day 1 (log_2_FC > 1, padj < 0.01). This approach identified 1,163 potential TET1 direct targets in miR-124 + ISX9-iNs on day 7.

A similar approach was followed for the identification of LIN28A direct targets, where we used publicly available data from a FLAG-tagged LIN28A-ChIP-seq experiment performed in ESCs [63]. We used only the genes in which LIN28A was found to bind to their TSS or proximal promoter (-/+ 1 kb from the TSS) and filtered them for genes that were upregulated in miR-124 + ISX9 day 7 vs astro day 1 (log_2_FC > 1, padj < 0.01). This approach led to 238 potential LIN28A direct targets in miR-124 + ISX9-iNs on day 7.

### Immunocytochemistry

The cells were washed once with PBS and then fixed with 4% paraformaldehyde for 20 min at room temperature. Afterwards, the cells were washed three times with PBS and blocked with 5% normal donkey serum (NDS) (Merck-Millipore) and 0.1% Triton X-100 in PBS for 1 h at room temperature. Next, the cells were incubated with primary antibodies diluted in 1% NDS and 0.05% Triton X-100 in PBS overnight at 4 °C. The next day, the cells were washed three times with PBS and incubated with secondary antibodies diluted in 1% NDS or 0.05% Triton X-100 in PBS for 2 h at room temperature. The nuclei of the cells were stained with Everbrite mounting medium with DAPI (Biotium) or DAPI (Biotium) and Mowiol (Sigma) as the mounting medium. The following primary antibodies were used in this study: mouse anti-TUJ1 (BioLegend, 1:600), rabbit anti-TET1 (Abcam, 1:1000), rabbit anti-LIN28A (Proteintech, 1:150), mouse anti-MAP2 (Millipore, 1:400), rabbit anti-SYNAPSIN1 (Abcam, 1:200) and rabbit anti-SYNAPTOPHYSIN (Cell Signaling, 1:100). The secondary antibodies used in this study were Alexa Fluor 546-, Alexa Fluor 488- and Alexa Fluor 647-conjugated secondary antibodies (Life Technologies). Images were acquired with a 40 × objective (1024 × 1024 pixels, 1 μm Z-step) using a Leica TCS SP8 confocal microscope (Leica Microsystems). For each experiment, measurements from approx. 25 fields per coverslip were obtained for each condition.

### Image analysis

#### Morphological Characterization of TUJ1 + Cells Undergoing Reprogramming Using ImageJ Software

Morphological characterization of cells positively stained for the neuronal marker TUJ1 was conducted using Fiji/ImageJ software (National Institutes of Health). More specifically, TUJ1 + cells analyzed at days 5–11 of reprogramming were classified as follows. Initially, the soma size of TUJ1 + cells was calculated using the polygon selections tool to demarcate the soma and define the area size. TUJ1 + cells with an astrocyte-like morphology, which harbored a very large soma (the area of the soma ranged from 1000–1600 a.u) and exhibited none or very few processes were excluded from the analysis (representative cells are shown in Suppl. Figure 3D). The remaining TUJ1 + cells were considered to undergo reprogramming and were divided into three categories based on their soma size and the shape of their primary neurites. The first category included TUJ1 + cells that exhibited the most differentiated morphology defined by a small round soma (the area of the soma ranged from 85–200 a.u.) and well-shaped primary neurites (representative cells of category 1 are shown in Suppl. Figure  3 A). These cells were considered to undergo successful reprogramming. The second category comprised TUJ1 + cells that exhibited a larger soma (the area of the soma ranged from 200–500 a.u.) harboring mainly well-shaped, but also containing none or a few still not well-defined primary neurites (representative cells of category 2 are shown in Suppl. Figure 3B). These cells were considered as progressing efficiently in the reprogramming process, less successfully though than the cells of category 1. The third category consisted of TUJ1 + cells that possessed a large soma (the area of the soma ranged from 500–1000 a.u.) harboring a few well shaped and/or not well shaped primary neurites. These cells were considered as undergoing reprogramming, however less efficiently and in a retarded mode (representative cells of category 3 are shown in Suppl. Figure  3 C). The first two categories were considered as differentiated TUJ1 + cells, while the third one as TUJ1 + cells with a transitory morphology, still not sufficiently reprogrammed. For each experiment, measurements from at least 300–500 cells were obtained for each condition from 3 independent biological experiments.

#### Mean Fuorescence Intensity Measurement of TET1 and LIN28A Immunostaining Using Imaris Software

In order to estimate the protein levels of TET1 and LIN28A at the single cell level we measured the mean fluorescence intensity (f.i.) of the green channel inside the cell nuclei using Imaris 10.2 image analysis software for both TET1 and LIN28A immunofluorescence stainings. More specifically, a surface was created for the detection of the nuclei (stained with DAPI) and the mean f.i. of the green channel was measured inside this surface.

Specifically for the LIN28A staining, the mean f.i. was also measured in the total cell body and the cytoplasm in addition to the nucleus, since LIN28A functions both in the nucleus and the cytoplasm. For this, a surface was created including the cell bodies (and in some cases part of the primary neurites) and used for the measurement of the mean f.i. of LIN28A (total mean f.i.).

Following, for the measurement of the mean f.i. of LIN28A only in the cytoplasm, a new channel was created from the subtraction of the nuclei channel (stained with DAPI) from the LIN28A channel. In this way, the new channel included only the cytoplasmic staining. Subsequently, the cytoplasmic mean f.i. of LIN28A was measured using a surface created with the same parameters that were used for the creation of the previously described surface developed for the estimation of the total mean f.i. of LIN28A.

#### Morphological Characterization of iNs Using the Filament Tracer Module in Imaris Software

Individual cell isolation for deeper morphological characterization of iNs was performed using the Imaris software (v.9.3.1, Oxford Instruments) extracting only the cells whose entire cell body and fine processes were included in the 3D stack. Initially, a new channel including the gray (TUJ1 staining), green (SYN1 staining) and magenta (MAP2 staining) channels was created, using the channel arithmetics module, in order to get all the possible morphological information from all fluorescence stainings (since not all iNs are at the same level of differentiation). This new channel was subsequently used for the 3D reconstruction of individual cells with the Filament Tracer module of Imaris. Briefly, the Filament Tracer locates a sphere (cell body) as the beginning point and reconstructs the processes as either main or secondary branches. The cell bodies were located after a 10 μm sphere was set as the beginning point, which reflects the average astrocyte cell body diameter. All the cells were analyzed using the same settings, including thresholding and the rest adjusted parameters. For each individual cell analyzed, Imaris provides various morphological properties.

The complexity and degree of ramification were assessed via Sholl analysis [79]. The number of intersections of iNs’ processes with concentric Sholl spheres at increasing distances from the soma was calculated for an increment of 1 μm from the cell body. The number of dendrite (primary neurite) branching points was also calculated for each analyzed cell, indicating the points where the main process splits into a secondary branch.

In addition, semiautomatic dendrite detection was performed for all the analyzed cells, using Filament tracer to measure dendrite (primary neurite) length.

### Statistical Analysis

All in vitro quantified data are presented as average ± SD unless otherwise indicated. A two-tailed t test assuming unequal variance was used to calculate statistical significance with p values, for all the data obtained in this study, where p values less than 0.05 (p < 0.05) were considered indicative of significance. Sholl analysis distributions were compared with a two-sample Kolmogorov‒Smirnov test.

## Supplementary Information

Below is the link to the electronic supplementary material.

Supplementary file1 (PDF 32121 KB)

Supplementary file2 (DOCX 14 KB)

## Data Availability

No datasets were generated or analysed during the current study.
