# Supplementary material for: The DNA Demethylase TET1 is a Pivotal Regulator of the miR-124/ISX9-Instructed Conversion of Astrocytes to Induced Neurons
Source: Mol Neurobiol. 2026 Apr 25;63(1):586. doi: 10.1007/s12035-026-05873-1 (PMC13110216; doi:10.1007/s12035-026-05873-1)
Supplement: Supplementary file 1 — Supplementary file1 (PDF 32121 KB) [file 12035_2026_5873_MOESM1_ESM.pdf]

Supplementary information

DNA demethylase TET1 emerges as a pivotal regulator of the miR-124/ISX9-instructed conversion of astrocytes to induced-neurons

Elsa Papadimitriou[1,#], Lukas daCC Iohan[2], Alexandra Frazeskou[1], Evangelia Xingi[3], Marcos R Costa[2,4], Dimitra Thomaidou[1,3,#]

Suppl. Figure 1

A

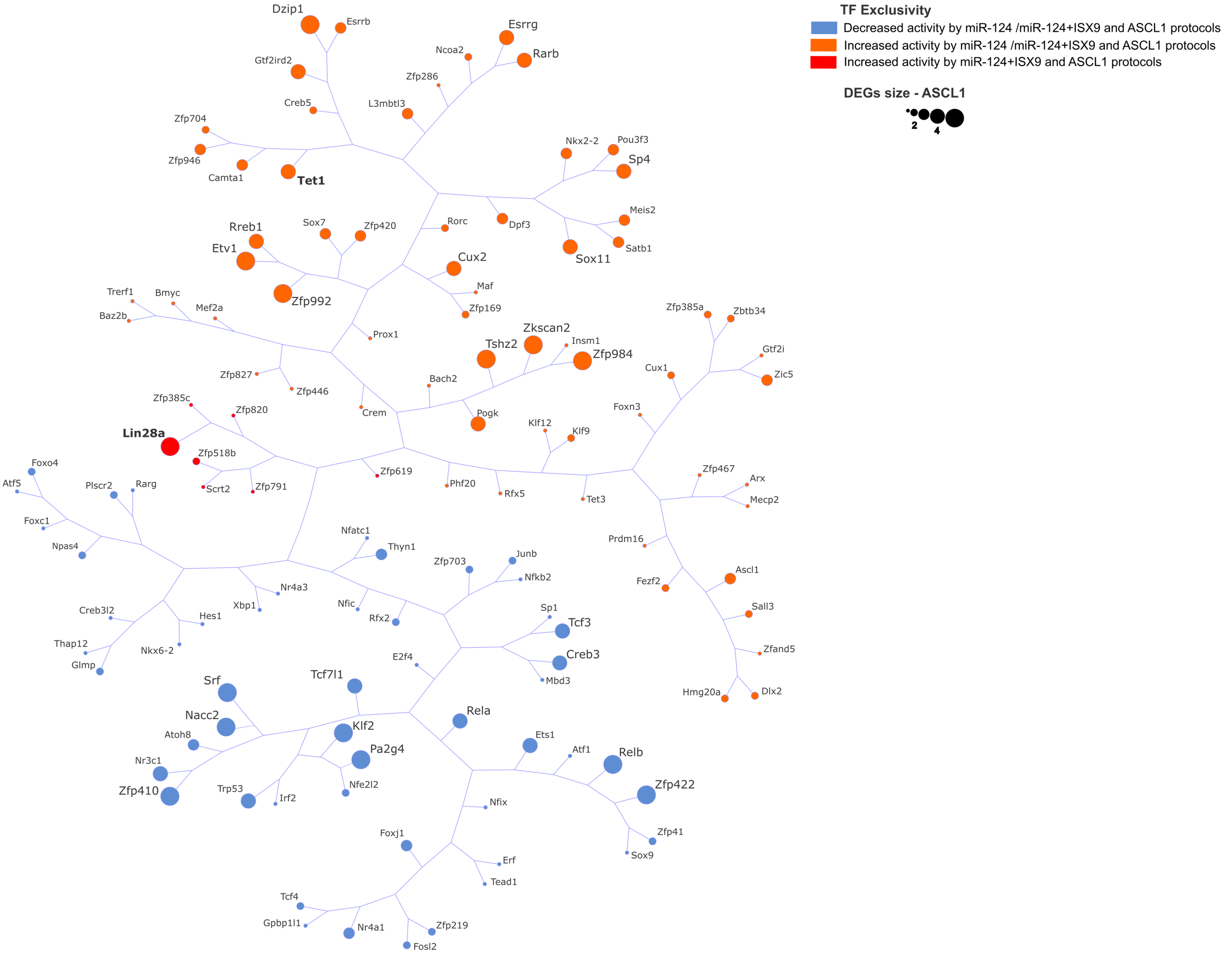

B

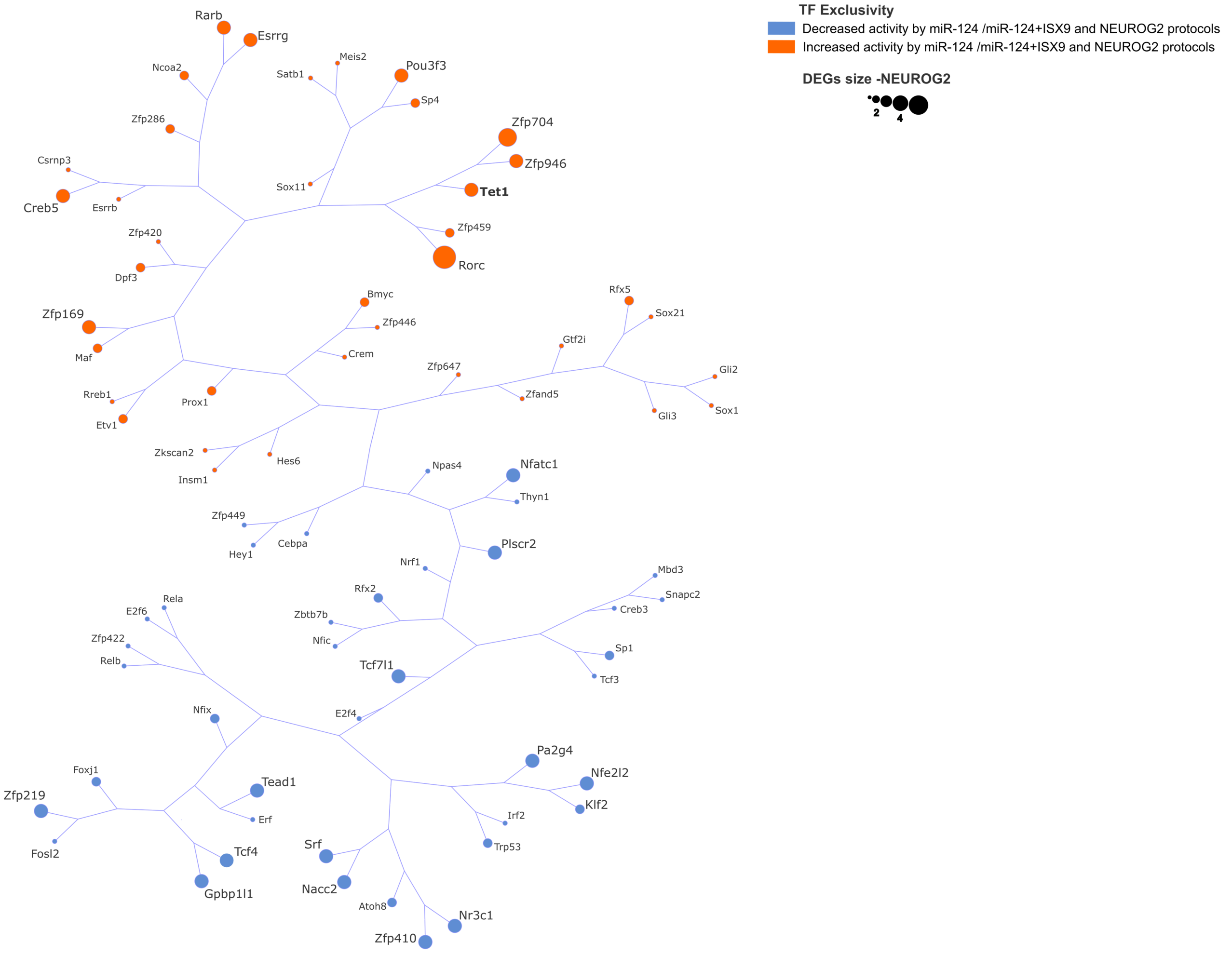

Supplementary Figure 1 (related to Figure 1)

Transcriptional regulatory networks showing common regulons identified in miR-124-iNs / miR-124+ISX9-iNs and ASCL1-iNs **(A)** and miR-124-iNs /miR-124+ISX9-iNs and NEUROG2-iNs **(B)**. Colors indicate regulons with decreased (blue) or increased (orange) activity during the astrocyte-to-neuron conversion between all protocols or increased (red) activity between miR-124+ISX9 and ASCL1 reprogramming protocols.

Suppl. Figure 2

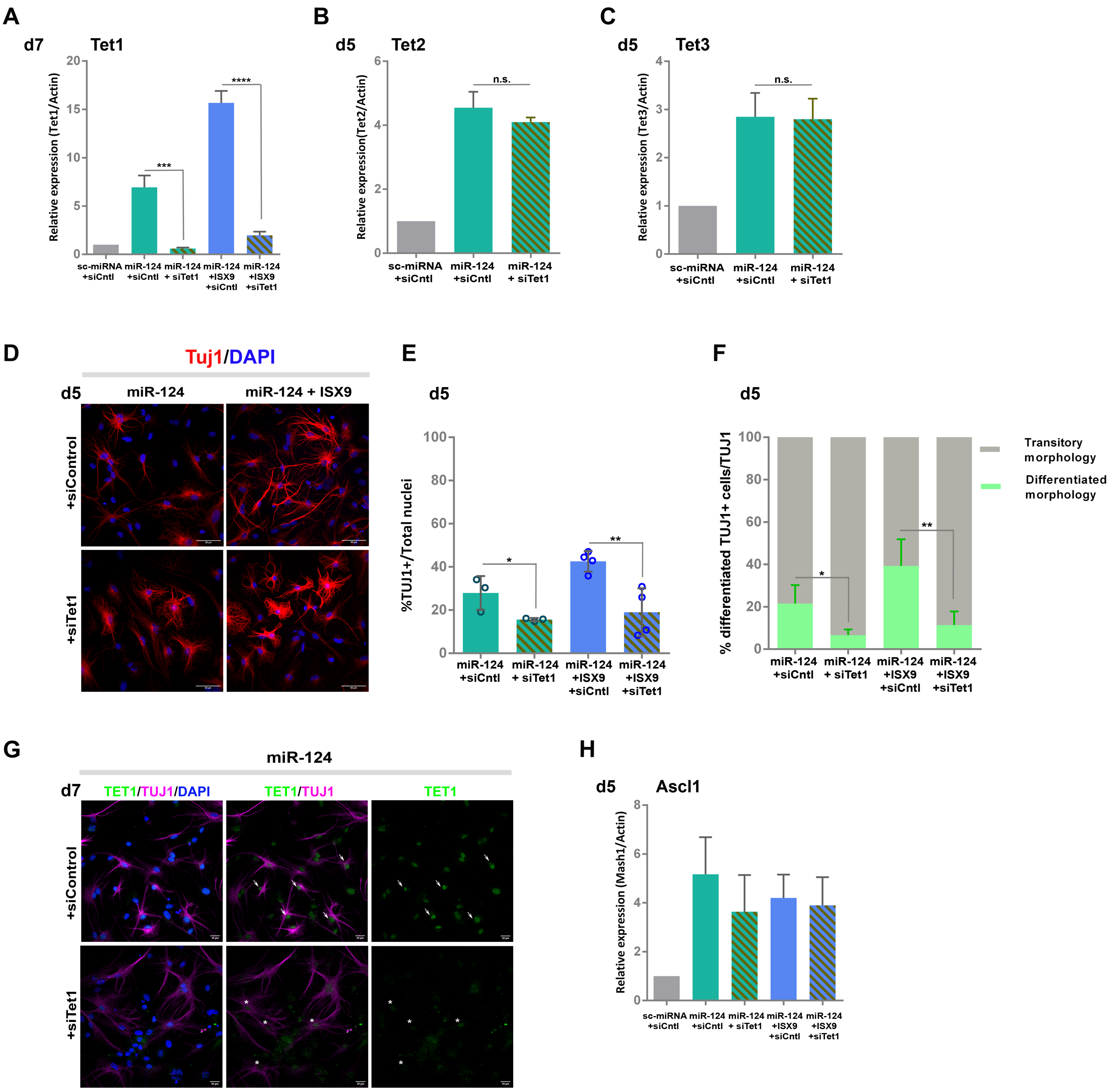

Supplementary Figure 2 (related to Figure 2)

A. Estimation of the silencing degree of *Tet1* mRNA levels on day 7 of reprogramming by miR-124 -/+ siTet1 or miR-124+ISX9 -/+ siTet1 (n=3) by qRT-PCR.

Estimation of the effect of *Tet1* silencing on the mRNA levels of *Tet2* (**B**) and *Tet3* (**C**) on day 5 of reprogramming by miR-124 -/+ siTet1 (n=3) by qRT-PCR.

D. Immunostaining of astrocytes reprogrammed with miR-124 -/+ siTet1 or miR-124+ISX9 -/+ siTet1 on day 5 with an anti-TUJ1 antibody (in red).

E. Quantification of the percentage of TUJ1+ reprogrammed cells with miR-124 -/+ siTet1 (n=3 independent experiments) or miR-124+ISX9 -/+ siTet1 (n=4 independent experiments) on day 5.

F. Presentation of the proportion of differentiated TUJ1+ iNs (green portions of the bars) in the total TUJ1+ population (as quantified in **E** for each condition and set to 100%) (the gray portions of the bars indicate the proportion of TUJ1+ iNs exhibiting a transitory still not differentiated morphology).

G. Immunostaining of astrocytes reprogrammed with miR-124 -/+ siTet1 on day 7 with an anti-TUJ1 antibody (in magenta) and an anti-TET1 antibody (in green); representative cells exhibiting high TET1 levels and a differentiated morphology (category 2) are indicated with arrows, while representative cells with low TET1 and low TUJ1 levels (category 3) are indicated with asterisks.

H. RT-qPCR analysis of the mRNA levels of the proneural TF *Ascl1* at day 5 of reprogramming by miR-124 -/+ siTet1 or miR-124+ISX9 -/+ siTet1 (n=3).

***p<0.001, ****p<0.0001.

Suppl. Figure 3

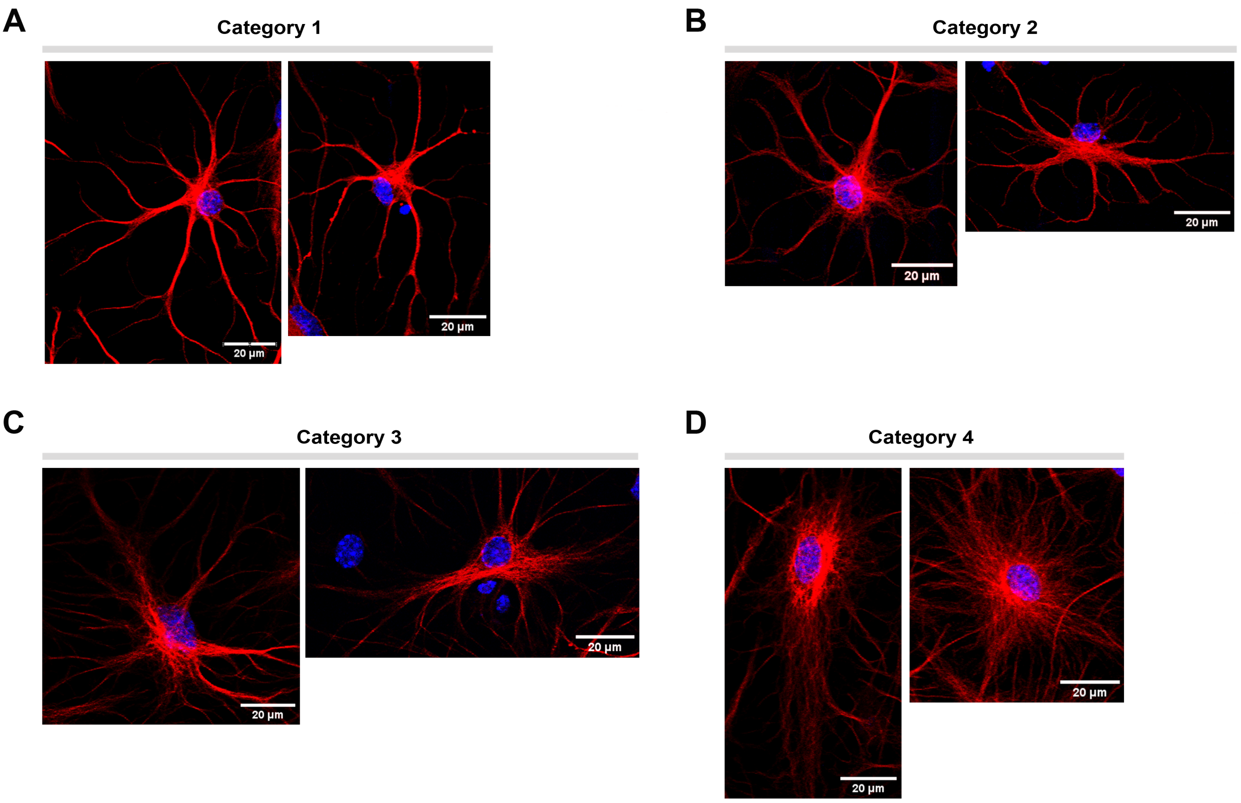

Supplementary Figure 3 (related to Figures 2, 3 and 5)

Representative cell morphologies of TUJ1+ cells belonging to the categories 1-4 used for the morphological analyses presented in Figures 2, 3 and 5

A. Representative TUJ1+ cells of category 1 exhibiting soma areas ranging from 85 to 200 a.u. and fully developed primary neurites. These cells are considered to undergo successful reprogramming and present the most differentiated morphology by day 7.

B. Representative TUJ1+ cells of category 2 exhibiting soma areas ranging from 200 to 500 a.u. harboring mainly well-shaped, but also containing none or a few still not well-defined primary neurites. These cells were considered as progressing efficiently in the reprogramming process, less successfully though than the cells of category 1.

C. Representative TUJ1+ cells of category 3 exhibiting soma areas ranging from 500 to 1000 a.u. harboring a few well shaped and/or not well shaped primary neurites. These cells were considered as still undergoing reprogramming, however less efficiently than the other two categories.

D. Representative TUJ1+ cells of category 4 exhibiting soma areas ranging from 1000 to 1600 a.u. harboring a very large soma and exhibiting none or very few processes. These cells were considered as astrocyte-like cells that failed to undergo reprogramming.

Suppl. Figure 4

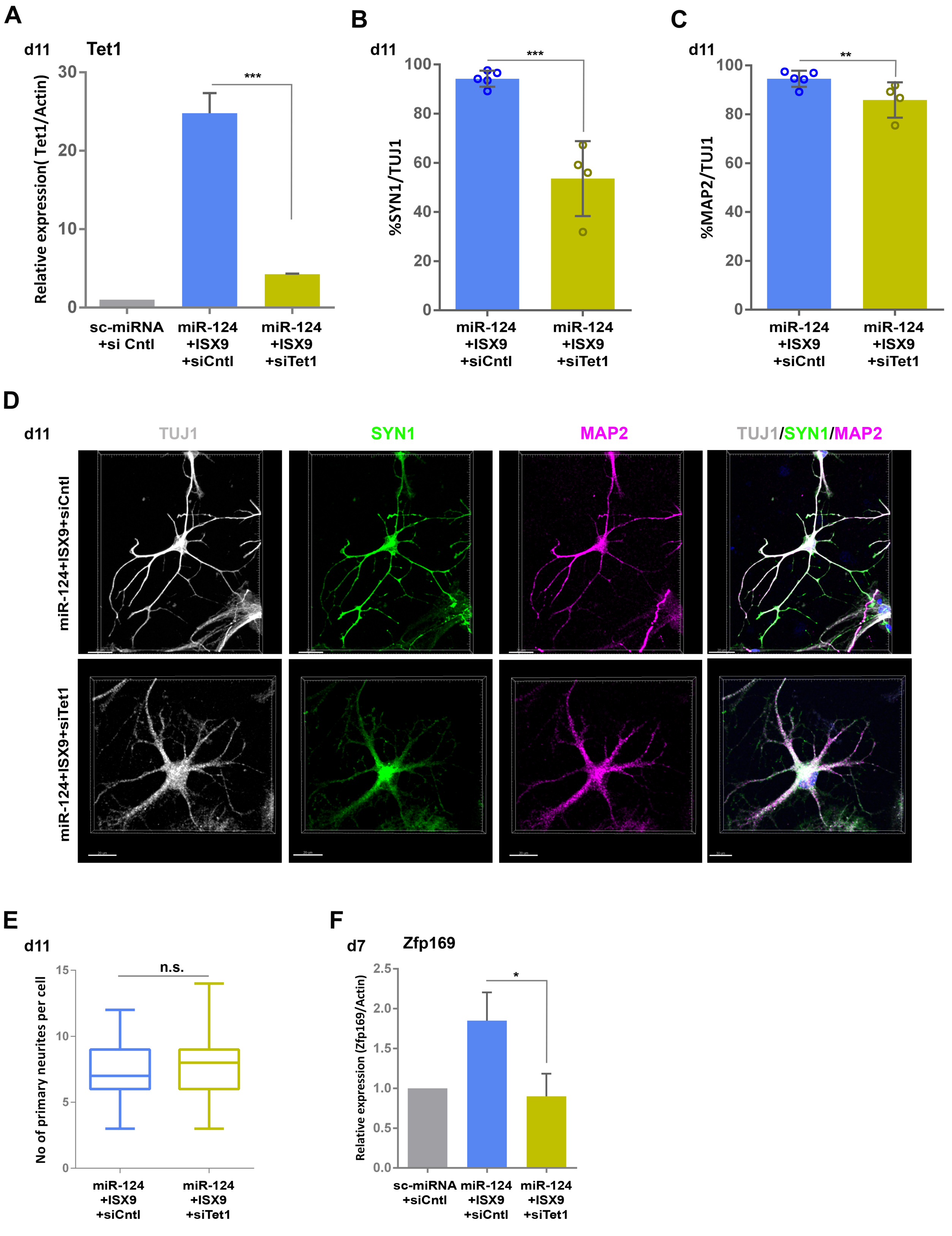

Supplementary Figure 4 (related to Figure 3)

A. Estimation of the silencing degree of *Tet1* mRNA levels on day 10 of reprogramming by miR-124+ISX9 -/+ siTet1 (n=3) by qRT-PCR.

B. Quantification of the proportion of the TUJ1+ iNs reprogrammed by miR-124+ISX9+siCntl (n=5) or miR-124+ISX9+siTet1 (n=4) that are also positive for SYN1 at day 11.

C. Quantification of the proportion of the TUJ1+ iNs reprogrammed by miR-124+ISX9+siCntl (n=5) or miR-124+ISX9+siTet1 (n=4) that are also positive for MAP2 at day 11.

D. Confocal images of the miR-124+ISX9+siCntl-iN and miR-124+ISX9+siTet1-iN co-stained for TUJ1 (in gray), SYN1 (in green) and MAP2 (in magenta) that are presented in **Figure 3E** after being processed by the Filament Tracer module in Imaris.

E. Quantification of the number of primary neurites per cell in miR-124+ISX9+siCntll-iNs (n=300) and miR-124+ISX9+siTet1-iNs (n=224) in ImageJ.

F. RT-qPCR analysis of the mRNA levels of the TF *Zfp169*, identified as part of TET1 regulon, on day 7 of reprogramming by miR-124+ISX9 -/+ siTet1 (n=3).

*p<0.05, **p<0.01, ***p<0.001.

Suppl. Figure 5

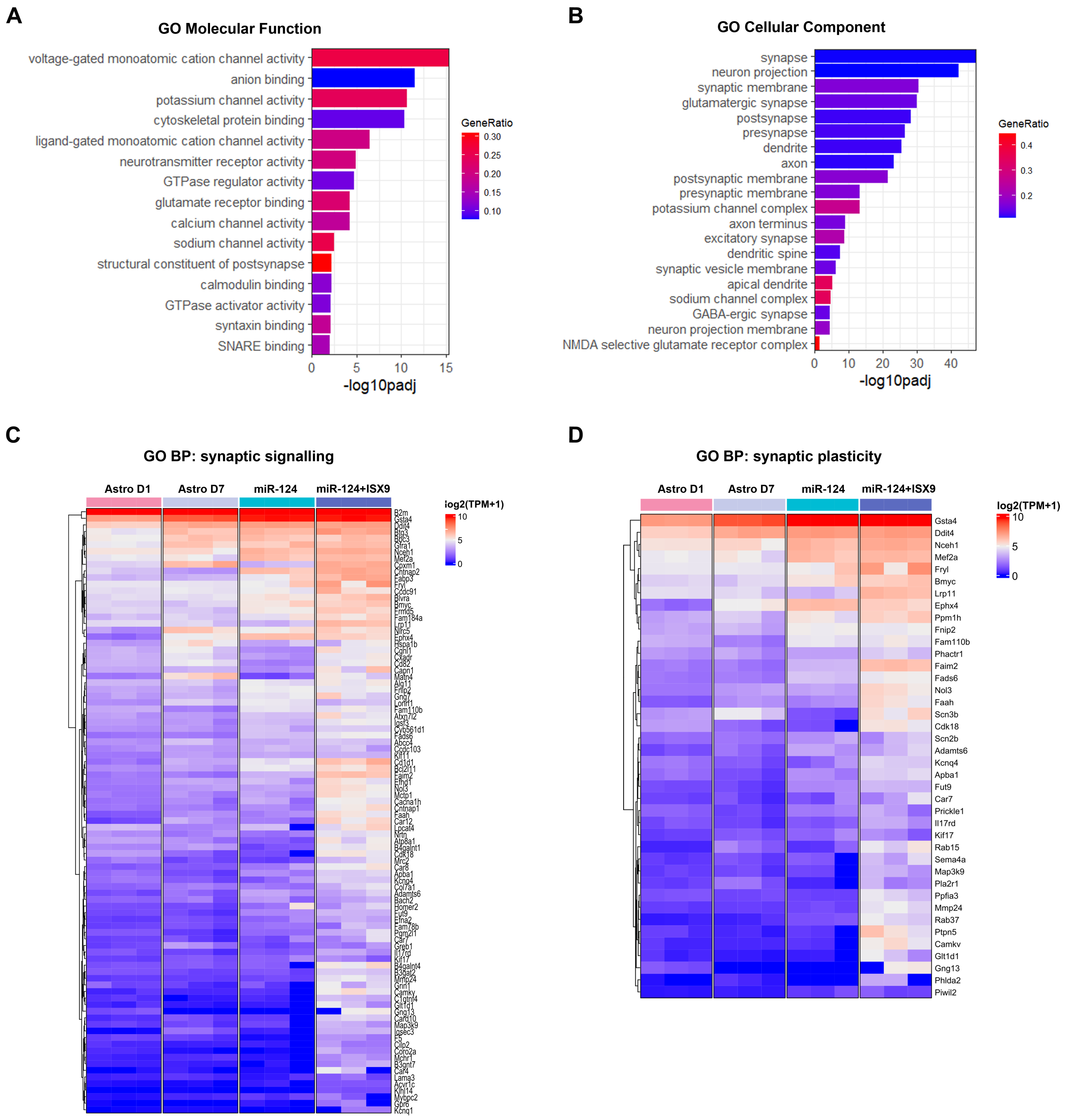

Supplementary Figure 5 (related to Figure 4)

Gene Ontology (GO) of Molecular Functions (MF) (**A**) and Cellular Components (CC) (**B**) terms enriched for the 1,163 TET1 direct targets up-regulated in miR-124+ISX9-iNs at day 7. GO terms are ranked by the p adjusted values and color coded by the gene ratio (the number of genes in the imputed gene set divided by the total number of genes in the GO term). Heat maps showing the expression of genes that belong to the GO BP "synaptic signaling" (**C**) and "synaptic plasticity" (**D**).

Suppl. Figure 6

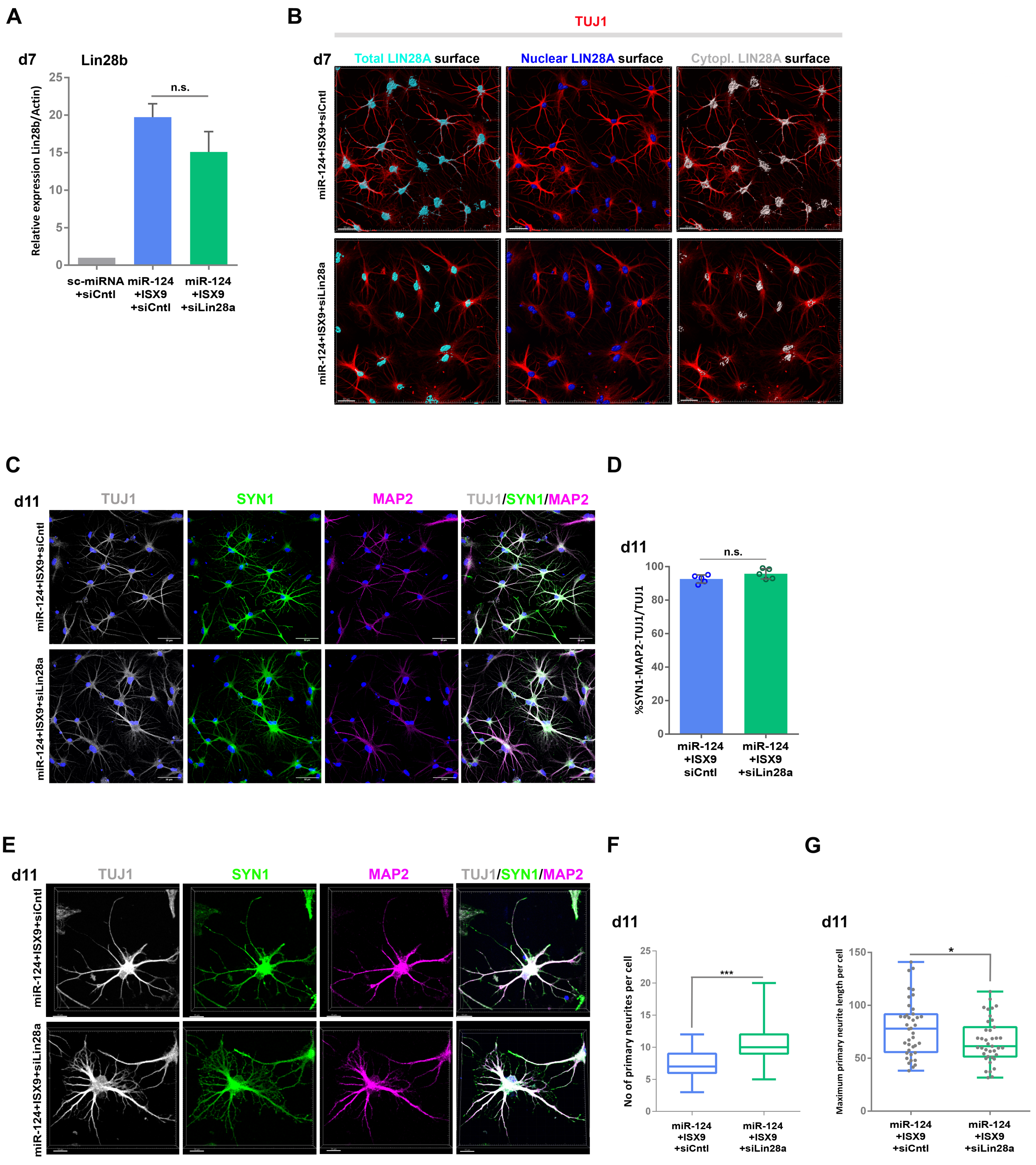

Supplementary Figure 6 (related to Figure 5)

A. Estimation of the effect of *Lin28a* silencing on the mRNA levels of *Lin28b* on day 7 of reprogramming by miR-124+ISX9 -/+ siLin28a (n=3) by qRT-PCR.

B. Processed images by Imaris corresponding to the confocal images presented in **Figure 5F**, showing the total (in cyan blue), nuclear (in blue) and cytoplasmic (in gray) surface along with TUJ1 staining (in red) that was created for the estimation of the total, nuclear and cytoplasmic levels of LIN28A by measuring the mean fluorescence intensity of LIN28A channel within each surface.

C. Coimmunostaining of astrocytes reprogrammed with miR-124+ISX9 -/+ siLin28a on day 11 with anti-TUJ1 (in gray), anti-SYN1 (in green) and anti-MAP2 (in magenta) antibodies.

D. Quantification of the percentage of TUJ1+ iNs reprogrammed by miR-124+ISX9+siCntl or miR-124+ISX9+siLin28a that were also positive for SYN1 and MAP2 on day 11 (n=5 independent experiments).

E. Confocal images of the miR-124+ISX9+siCntl-iN and miR-124+ISX9+siLin28a-iN costained for TUJ1 (in gray), SYN1 (in green) and MAP2 (in magenta) that are presented in **Figure 5J** after being processed by the Filament Tracer module in Imaris.

F. Quantification of the number of primary neurites per cell in miR-124+ISX9+siCntl-iNs (n=300) and miR-124+ISX9+siLin28a-iNs (n=243) in ImageJ.

G. Quantification of the maximum primary neurite length per cell in miR-124+ISX9+siCntl-iNs (n=40 cells) and miR-124+ISX9+siLin28a-iNs (n=39 cells) using the Filament Tracer module in Imaris. The cells that were analyzed with the Filament Tracer module in Imaris were collected from 3 independent experiments.

*$p<0.05$, ***$p<0.001$.

Suppl. Figure 7

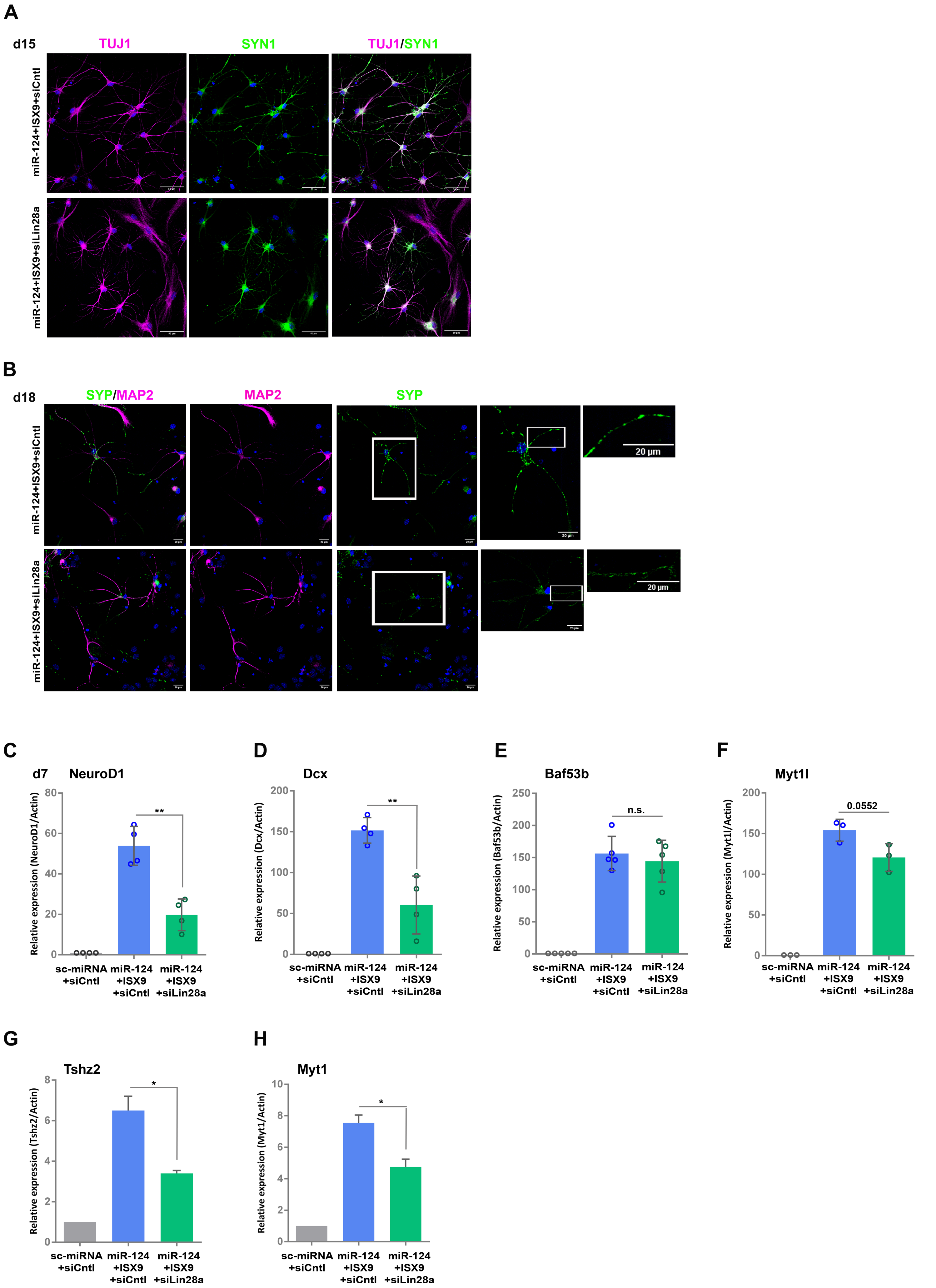

Supplementary Figure 7 (related to Figure 5)

A. Co-immunostaining of reprogrammed iNs by miR-124+ISX9 -/+ siLin28a on day 15 with anti-TUJ1 (in magenta) and anti-SYN1 (in green).

B. Coimmunostaining of miR-124+ISX9+siCntl-iNs and miR-124+ISX9+siLin28a-iNs on day 18 with an anti-MAP2 antibody (in magenta) and an anti-SYP antibody (in green); the inset areas show higher magnifications of representative cells and their processes stained with SYP (Synaptophysin).

RT-qPCR analysis of the mRNA levels of *NeuroD1* (**C**), *Dcx* (**D**), *Baf35b* (**E**) and *Myt1l* (**F**) on day 7 of reprogramming by miR-124+ISX9 -/+ siLin28a (n=3).

RT-qPCR analysis of the mRNA levels of the TFs Tshz2 (**G**) and Myt1 (**H**), identified as part of LIN28A regulon, on day 7 of reprogramming by miR-124+ISX9 -/+ siLin28a (n=3).

*$p<0.05$, **$p<0.01$, ***$p<0.001$.

Suppl. Figure 8

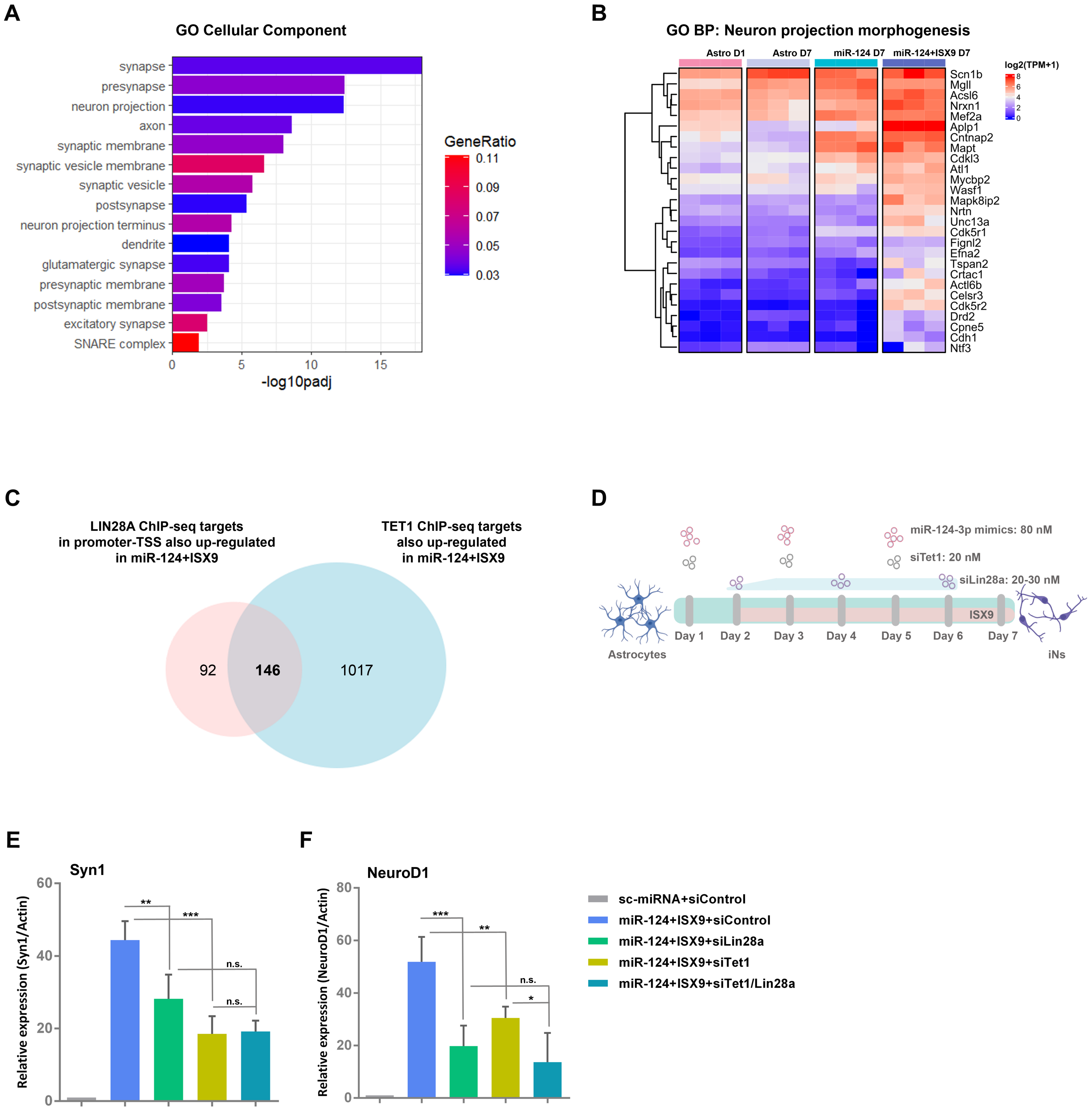

Supplementary Figure 8 (related to Figure 6)

A. Gene Ontology (GO) of Cellular Components (CC) terms enriched for the 238 LIN28A direct targets up-regulated in miR-124+ISX9-iNs at day7. GO terms are ranked by the p adjusted values and color coded by the gene ratio (the number of genes in the imputed gene set divided by the total number of genes in the GO term).

B. Heat map showing the expression of genes that belong to the GO BP "neuron projection morphogenesis".

C. Venn diagram representing the overlap between the LIN28A direct targets also up-regulated in miR-124+ISX9-iNs at day 7 and TET1 direct targets also up-regulated in miR-124+ISX9-iNs at day 7, resulting in 146 genes.

D. Schematic representation of the protocol that was followed for the cosilencing of *Tet1* and *Lin28a* in reprogrammed astrocytes by miR-124+ISX9.

RT-qPCR analysis of the mRNA levels of the genes *Syn1 (***E**) and *NeuroD1* (**F**) on day 7 of reprogramming in miR-124+ISX9+siCntl, miR-24+ISX9+siLin28a, miR-124+ISX9+siTet1 or miR-124+ISX9+siTet1/Lin28a.

**p<0.01, ***p<0.001.

Supplementary File 1: Excel file presenting the regulon activity scores inferred by the RTN analysis and the activity scores of top 40 regulons inferred by the betweenness centrality analysis

Supplementary File 2: Excel file presenting the betweenness centrality score for the regulons of the TRN

Supplementary File 3: Excel file presenting the TET1 and LIN28A regulons inferred by the RTN analysis
