# Supplementary material for: The DNA Demethylase TET1 is a Pivotal Regulator of the miR-124/ISX9-Instructed Conversion of Astrocytes to Induced Neurons
Source: Mol Neurobiol. 2026 Apr 25;63(1):586. doi: 10.1007/s12035-026-05873-1 (PMC13110216; doi:10.1007/s12035-026-05873-1)
Supplement: Supplementary file 2 — Supplementary file2 (DOCX 14 KB) [file 12035_2026_5873_MOESM2_ESM.docx]

**Table S1:** Sequences of the primers used for real-time PCR in this study

| Gene | Forward primer | Reverse primer |
| --- | --- | --- |
| Actin | CCCAGGATTGCTGACAGG | TGGAAGGTGGACAGTGAGGC |
| Baf53b | GTGTCTGATTCCCCTTACGATG | GACAGGCCCTTGACATTAGAG |
| Cacng2 | GACACCGCAGAGTATTTCCTC | TGTGGCGTGTCTTGTAGAAC |
| Celsr3 | GGACTTTGGAAATGACATGGC | CAGACAGCAACCCTCGATC |
| Dcx | CAGTCAGCTCTCAACACCTAAG | CATCTTTCACATGGAATCGCC |
| Insm1 | GCCACCCGTCTGAGAATAG | CTTTGTGGGTCTCCGAGTG |
| Kif5a | CATCAAGCAGGAGAACGTAGAG | TGATATTCTTTGCCTCGTCCAG |
| Lin28a | TGTTCTGTATTGGGAGTGAGC | GCTTGCATTCCTTGGCATG |
| Lin28b | GAGTCAATACGGGTAACAGGC | TTCTCGCACAGTCCACATC |
| Mash1 | TCTCCGGTCTCGTCCTACTC | CAAAGTCCATTCCCAGGAGA |
| Myt1 | AAGTTTGTCTGGCTGTCCC | GTCTCGTATCTCCTGATTTAGCTG |
| Myt1l | AGAGCAAAGAGGACAAGGAAG | TACCCATCTTTCTGCCTCTTG |
| NeuroD1 | TTGAAGCCATGAATGCAGAG | TCTTGGGCTTTTGATCATCC |
| Rab3c | TTCCTGTTCCGTTATGCCG | GATGGTCCTGTATCTTTCCTGG |
| Syt4 | TCCACTACAAACACGCTCAC | TGGTACAGGTTCACTTTGACG |
| Syn1 | GTGGATTCTCTGTGGACATGG | CAGCCCAATGACCAAACTTC |
| Scrt1 | AATCATGCCCAGGTCCTTC | CCACGTAGTCACTGAGGTATC |
| Tbr2 | CACCCAGAATCTCCTAACACTG | AGCCTCGGTTGGTATTTGTG |
| Tox | TGCTCTCCAATTCCATCTCTG | CTGTCTGATGTCTGTAGGCTG |
| Tet1 | GTCAGGGAGCTCATGGAGAC | CCTGAGAGCTCTTCCCTTCC |
| Tet2 | CCAGAAGCAAGAAACCAAGG | GAGCAATGACAGTAGCCAGG |
| Tet3 | AGACCCTTCTCAGGGGTCAC | GTGCAGTTGCTCGTCCTCAG |
| Zfp169 | GGATGCTTTCTGGACCTTTG | CTTGGAAAAGTCTGAGAGGGAG |
